# A Foxp2 Mutation Implicated in Human Speech Deficits Alters Sequencing of Ultrasonic Vocalizations in Adult Male Mice

**DOI:** 10.3389/fnbeh.2016.00197

**Published:** 2016-10-20

**Authors:** Jonathan Chabout, Abhra Sarkar, Sheel R. Patel, Taylor Radden, David B. Dunson, Simon E. Fisher, Erich D. Jarvis

**Affiliations:** ^1^Department of Neurobiology, Duke University Medical CenterDurham, NC, USA; ^2^Howard Hughes Medical InstituteChevy Chase, MD, USA; ^3^Department of Statistical Science, Duke UniversityDurham, NC, USA; ^4^Language and Genetics Department, Max Planck Institute for PsycholinguisticsNijmegen, Netherlands; ^5^Donders Institute for Brain, Cognition and Behavior, Radboud UniversityNijmegen, Netherlands; ^6^The Rockefeller UniversityNew York, NY, USA

**Keywords:** FoxP2, speech apraxia, ultrasonic vocalizations, song, syntax, KE family

## Abstract

Development of proficient spoken language skills is disrupted by mutations of the *FOXP2* transcription factor. A heterozygous missense mutation in the KE family causes speech apraxia, involving difficulty producing words with complex learned sequences of syllables. Manipulations in songbirds have helped to elucidate the role of this gene in vocal learning, but findings in non-human mammals have been limited or inconclusive. Here, we performed a systematic study of ultrasonic vocalizations (USVs) of adult male mice carrying the KE family mutation. Using novel statistical tools, we found that *Foxp2* heterozygous mice did not have detectable changes in USV syllable acoustic structure, but produced shorter sequences and did not shift to more complex syntax in social contexts where wildtype animals did. Heterozygous mice also displayed a shift in the position of their rudimentary laryngeal motor cortex (LMC) layer-5 neurons. Our findings indicate that although mouse USVs are mostly innate, the underlying contributions of FoxP2 to sequencing of vocalizations are conserved with humans.

## Introduction

Spoken language plays a central role in our culture and society, which we use to express emotions, convey ideas, and communicate. We belong to one of few species that learn to produce new vocalizations. These vocal behaviors are susceptible to a range of impairments, making dramatic impacts on our everyday life. Such deficits represent a major public health issue, with the prevalence of speech-sound disorder in young children estimated at 8–9% (NIDCD, 2010). These developmental speech and language disorders are highly heritable (Bishop et al., 1995), but the underlying causes remain elusive for most cases (Shriberg et al., 1999; Law et al., 2000).

In the past decade and a half, scientists have discovered that some spoken language disorders result from rare single-gene mutations. The most prominent example is a point mutation disrupting the *FOXP2* (forkhead-box P2) transcription factor in the KE family (Fisher et al., 1998; Lai et al., 2001, 2003). Affected individuals have difficulties mastering the coordinated movement sequences of syllables/phonemes for fluent speech, described as developmental verbal dyspraxia (DVD) or childhood apraxia of speech (CAS), as well as impacting written language. These deficits occur against a background of relatively preserved cognitive and physical abilities (Lai et al., 2001; Watkins et al., 2002a; Fisher et al., 2003). The affected KE family members carry a missense mutation in one copy of the *FOXP2* gene, yielding an arginine-to-histidine substitution (p.R553H) that disturbs the DNA-binding domain of the encoded protein (Fisher et al., 1998; Lai et al., 2001; Vernes et al., 2006). Subsequently, a growing number of other families and individuals with spoken language disorders have been identified with point mutations or chromosome rearrangements (translocations and deletions) involving the *FOXP2* gene (Bacon and Rappold, 2012; Turner et al., 2013).

Many downstream targets of the FOXP2 transcription factor control neural connectivity and plasticity (Fisher and Scharff, 2009), and functional experiments suggest a role in modulating neurite branching and length (Vernes et al., 2011). A number of FOXP2 target genes have been independently implicated in language impairments, autism, schizophrenia, bipolar disorders, epilepsy, and intellectual disabilities (Deriziotis and Fisher, 2013; Graham and Fisher, 2013). Comparative studies across vertebrates showed that FOXP2's coding sequence and brain expression are remarkably conserved (Lai et al., 2003; Haesler et al., 2004; Teramitsu et al., 2004). It is expressed in cortical and subcortical brain structures that are important for multimodal sensory processing, sensorimotor integration, and motor-skill learning (Lai et al., 2003). These include corticostriatal, corticocerebellar, sensory thalamic (Haesler et al., 2004; Teramitsu et al., 2004), and midbrain modulatory circuits (Campbell et al., 2009) involved in the acquisition and performance of motor skills (Ferland et al., 2003; Lai et al., 2003; Campbell et al., 2009). It has been hypothesized that the ancestral ortholog in mammals contributes to the development of motor-related brain regions (Lai et al., 2003; Fisher and Marcus, 2006; Fujita and Sugihara, 2012; Garcia-Calero et al., 2016). Moreover, manipulations of the avian ortholog (*FoxP2*) in the songbird striatal nucleus Area X have demonstrated *FoxP2's* role in vocal learning and plasticity, during development and adulthood (Haesler et al., 2007; Murugan et al., 2013).

**Figure 1:**
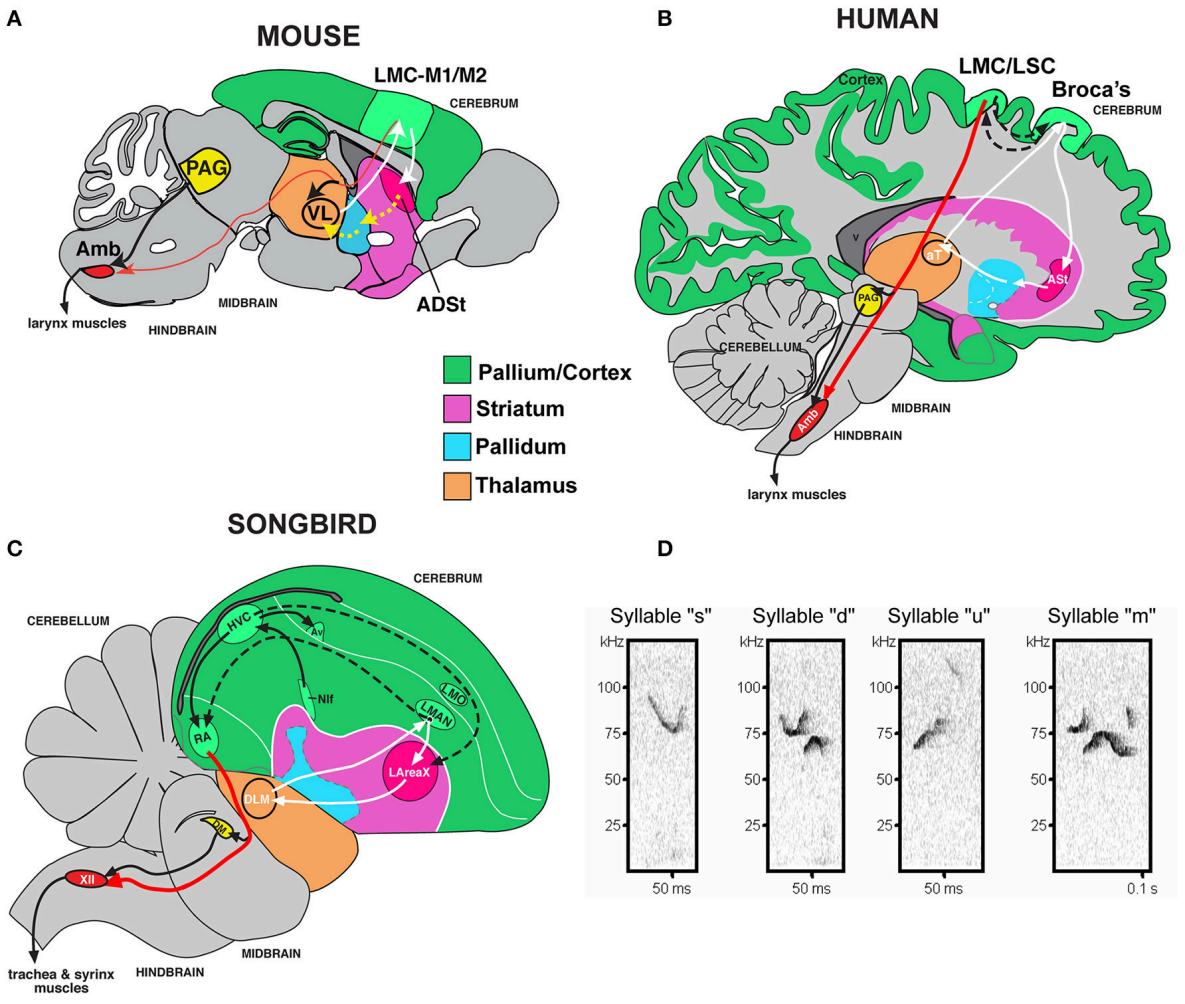
**Mouse song system anatomy and syllable types. (A)** Proposed anatomy of the rudimentary mouse forebrain vocal communication circuit based on Arriaga et al. (2012). Not shown are other connected brainstem regions, the amygdala, and insula. **(B)** Comparison with human, based on Arriaga et al. (2012) and Pfenning et al. (2014). **(C)** Comparison with songbird. **(D)** Sonograms of examples syllables of the four syllable categories quantified from a C57 male mouse USV song, labeled according to pitch jumps. Anatomical abbreviations: ADSt, anterior dorsal striatum; Amb, nucleus ambiguous; ASt, anterior striatum; aT, anterior thalamus; Av, nucleus avalanche; HVC, a letter-based name; LArea X, lateral Area X; LMO, lateral mesopallium oval nucleus; LMAN, lateral magnocellular nucleus of the nidopallium; LMC, laryngeal motor cortex; LSC, laryngeal somatosensory cortex; M1, primary motor cortex; M2, secondary motor cortex; NIf, interfacial nucleus of the nidopallium; PAG, periaqueductal gray; RA, robust nucleus of the arcopallium; T, thalamus; VL, ventral lateral nucleus of the thalamus; XIIts, 12th vocal motor nucleus, tracheosyringeal part.

It would be useful to know if a mammalian model could be used to study genetic deficits associated with language disorders. However, unlike human speech and learned birdsong, most species are vocal non-learners; this includes mice, where males have been reported to have limited to no plasticity of their ultrasonic vocalization (USV) songs (Grimsley et al., 2011; Kikusui et al., 2011; Arriaga et al., 2012; Hammerschmidt et al., 2012; Arriaga and Jarvis, 2013; Mahrt et al., 2013; Portfors and Perkel, 2014). Despite this limitation, rudimentary cortical-striatal circuits similar to those that control production of learned vocalizations in humans and songbirds are activated in the brains of adult mice when they vocalize (Arriaga et al., 2012). These circuits include an elementary laryngeal motor cortex (LMC) region of the mouse primary motor cortex (M1), once thought to be present only in humans among mammals, that makes a sparse projection (as opposed to dense projection in humans and songbirds) to motor neurons that control the vocal organ (larynx in mammals; syrinx in birds; Figures 1A–C; Arriaga et al., 2012; Okobi et al., 2013). In contrast to humans and the analogous forebrain region in song-learning birds, the LMC in mice is not necessary for producing normal vocalizations; nonetheless, it seems to be involved in modulating the frequency of USVs (Arriaga et al., 2012) [although this is debated from studies in genetically modified mice lacking most of the cortex (Hammerschmidt et al., 2015)].

Several mouse lines have been developed carrying *Foxp2* disruptions (French and Fisher, 2014). Mice with homozygous *Foxp2* disruptions display reduced postnatal weight gain, severe developmental delays, motor problems, and die at 3–4 weeks of age, demonstrating that *Foxp2* is necessary for long-term survival. Indeed, no human has been found with a homozygous inactivating *FOXP2* mutation. Shu et al. (2005) reported that heterozygous knockout pups emitted fewer ultrasonic isolation calls compared to wildtypes, along with moderate developmental delays. In contrast, other studies on mice with heterozygous *Foxp2* disruptions of various kinds found no significant alteration of pup calls and overtly normal development (French et al., 2007; Groszer et al., 2008; Gaub et al., 2010; French and Fisher, 2014). For example, Groszer et al. (2008) studied heterozygous mice carrying the KE family mutation (referred to as *Foxp2-R552H*, since the murine protein is one amino-acid shorter than the human), and found that pups produce normal numbers of isolation and distress calls, with normal characteristics. Gaub et al. (2010) showed with a null *Foxp2* mutation, that even homozygous pups produce normal temporal patterns of vocalizations and clicks, but only at comparably low intensities. Notably, most prior studies focused primarily on pup calls.

We previously showed that adult male mice modify their syntax, including syllable sequence length, composition, and order, based on different stimuli and social contexts (Chabout et al., 2015). That study used techniques from the songbird field and dynamic syntax analysis to characterize mouse USVs. “Syntax” is used here in its broad definition in studies of animal communication, which differs from formal definitions applied in human linguistics. In characterizing animal vocalizations, “syntax” denotes the properties of an ordered, non-random, sequence of sounds, whether or not the sequences have meaning to the listening animals.

In the current study, we developed more advanced statistical tools to characterize the effects of the KE heterozygous *Foxp2*-*R552H* mutation on USVs syntax of adult male mice, in different social contexts. We found that, as in humans, the KE *Foxp2* heterozygous mutation in mice affects more the sequencing than the acoustic structure of vocalizations. Using transynaptic-tracing techniques, we also found that the mutation is associated with a posterior shift in the position of the LMC layer-5 neurons.

## Methods

All experimental protocols were approved by the Duke University Institutional Animal Care and Use Committee (IACUC).

### Animals

*Foxp2-R552H* mutant mice were bred from strains previously described (Groszer et al., 2008); heterozygous fertile males were paired with C57BL6/J females. The pairs were housed in regular plastic home cages at average temperature of 25°C and a 12-h light-dark cycle. Wood shaving served as bedding, water and food were available *ad libitum*. To avoid post mating in the postpartum estrus female and ensure a calm raising of the pups by the female, fathers were removed at the day of birth of the pups. Pups were sexed, tagged at weaning (postnatal day 22). A tail sample was also taken for genotyping purposes. Young males were then group housed blind to genotype.

### Genotyping

Mice were genotyped by polymerase chain reaction (PCR) using *Titanium Taq polymerase* and restriction digestion of genomic DNA from tail samples. The following primers were used: Foxp2_Forward 5′-GTTCCTCTGGACATTTCAAC-3′ and Foxp2_Reverse 5′-TGTGAGCATGCCTTTAGCTG-3′. PCR conditions were as follows: 95°C for 3 min (1 cycle), 95°C for 30 s (13 cycles), 65°C (−0.5°C/cycle) for 30 s (13 cycles), 68°C for 45 s (13 cycles), 95°C for 30 s (25 cycles), 58°C for 30 s (25 cycles), 68°C for 40 s (25 cycles), 68°C for 7 min (1 cycle). The 603 bp PCR products were digested overnight at 37°C with *HgaI* which yields fragments of 372 and 231 bp for the wildtype allele, while the mutant R552H allele remains undigested.

### Recording of vocalizations in different social contexts

Vocalizations were recorded from males when they were around 8–9 weeks old (young adults). For this first set of animals, a total of 19 adult male mice were recorded, 10 heterozygous, and 9 wildtypes. One wildtype animal did not sing at all in any condition and was thus removed from the study (10 het/8 WT). After an overnight experience with a sexually mature wildtype female, male mice were placed back in the same social housed cages (4/5 mice per cage) until the test day. The males were then removed from their cages, placed in a new cage and then singly habituated in the sound recording environment (15″ × 24″ × 12″ beach cooler with a tube for pumped air circulation input, no light and a hanging microphone, as a soundproof compartment (Arriaga et al., 2012; Chabout et al., in press) for 15 min. Although no recordings were made for this period, overall observation of the live audio recording on the computer monitor by Avisoft Recorder USG software showed no songs were emitted during the habituation. We then exposed the males to one of the four different social contexts to stimulate singing (Chabout et al., 2015): (1) Fresh female urine collected from at least two different wildtype females from two distinct grouped housed cages within minutes of exposure on a urine-dipped cotton tip placed inside the male's cage (UF); (2) awake and behaving adult wildtype female placed inside the cage (LF); (3) an anesthetized wildtype female placed on the metal lid of the cage (AF); and (4) an anesthetized adult wildtype male placed on the metal lid of the cage (AM). We modified our original abbreviations for these context descriptions (Chabout et al., 2015) to have a more consistent systematic naming: first characteristic of the context (U-urine; L-Live; A-Anesthetized) followed by sex of the stimulus animal (F-female; M-male). Exposure and recordings lasted for 5 min. The same mouse was exposed on 3 consecutive days to the same social context (either UF, LF, AF, or AM), but the identity of the stimulus (specific animal) was changed every day to ensure against a familiarity effect. Then the next week, the same mouse was exposed to a different stimulus following the same procedure. We repeated this for 4 consecutive weeks, where the order of stimulus was shuffled between weeks and genotypes such that each animal received a different stimulus, in order to normalize against any possible order effect. We tried to use females in pro-estrus or estrus (wide vaginal opening and pink surround) for the female stimuli when possible with the scheduled recordings. The anesthetized animals were anesthetized with ketamine/xylazine (100 and 10 mg/kg, respectively, intraperitoneally) and put on a heat pad outside of the test cage between recording sessions for at least 5 min. Between trials, the recording cages were cleaned with 1% Trifectant and water.

To replicate our key findings using a different population of *Foxp2-R552H* mice from the same founder line, a total of 31 mice were recorded, 16 heterozygous and 15 wildtype. Males were treated the same as above, except that, for litter delivery reasons all males were treated sequentially in the three contexts in the following order: UF, AF, and LF. In this second experiment, the timing of litter deliveries of different males on different days did not allow us to randomize the study with groups of the same age or a maximum of 1 week apart. We still managed to test equal numbers of heterozygous and wildtype on test days. The first experiment above was conducted in October and the replicate experiment in March.

Sounds were recorded with UltraSoundGate CM16/CMPA ultrasound microphones that were suspended over the center of each cage in the recording box, high enough so that the receiving angle of the microphone covered the whole area of the test cage. The microphones were connected to a multichannel ultrasound recording interface Ultrasound Gate 416H, which was plugged into a computer running Avisoft Recorder USG software v4.2.18 (Sampling frequency: 250 kHz, FFT-length: 1024 points; 16-bits). All recording hardware and software were from Avisoft Bioacoustics® (Berlin, Germany). Further, detail of the recording method is described in Chabout et al. (in press).

### Acoustic definitions

Following standard definitions as described in Arriaga and Jarvis (2013), we considered a sound note as the most basic acoustic unit, formed by a single continuous sound with or without variations in fundamental frequency. One or more notes can be combined to form a “call” or a “syllable,” as a single acoustic unit not separated by silence. We distinguish “calls” and “syllables” by the pattern of usage. Calls are typically produced in isolation or in short bursts, and are usually repeated single acoustic unit types. Syllables, however, derive their classification from being included in a longer series of rapidly produced vocalizations of varying types. We define a sequence as a succession of syllables spaced by short intervals, with each sequence separated by a longer interval (250 ms or more) of silence as described in Chabout et al. (2015) and the main text. Thus, a song is a sequence of syllables, often elaborate, delivered periodically and sometimes with rhythm. When pitched to the human hearing range, male USV sequences in the four social contexts are strikingly reminiscent of the songs of certain songbirds (Holy and Guo, 2005; Arriaga et al., 2012).

### Sound analysis

Acoustic waveforms were processed using a custom MATLAB program (Arriaga et al., 2012), originally modified from code written by Timothy E. Holy (Holy and Guo, 2005) that we call “Mouse Song Analyzer v1.3” and is available on our website (http://jarvislab.net/research/mouse-vocal-communication/). Briefly, the software computed the sonograms from each waveform (256 samples/block, half overlap), thresholded to eliminate the white noise component of the signal, and truncated for frequencies outside the USV song range (35–125 kHz). We used a criterion of 10 ms minimum of silence to separate two syllables and 3 ms as the minimum duration of a syllable. The identified syllables were then classified by presence or absence of instantaneous “pitch jumps” separating notes within a syllable into four categories: (1) simple syllables without any pitch jumps (type “s”); (2) complex syllables containing two notes separated by a single upward (type “u”) or (3) downward (type “d”) pitch jump; and (4) more complex syllables containing two or more multiple pitch jumps (type “m”; Figure 1B). Any sounds that the software could not classify were put into an “unclassified” category and removed from the analysis. Manual visual inspection of the sonograms of the unclassified sounds revealed that most of them were either syllables that overlapped with mechanical, non-vocal noises the mouse made, such as scratching, walking on the plastic cage, chewing on the cage lid etc., or non-vocal mechanical sounds that included frequencies that reached above our 25 kHz cut off. All analyses were conducted on a total of 10,720 classified syllables in the urine condition (UF), 19,193 syllables in the anesthetized female condition (AF), 41,209 in the live female condition (LF), and 1,293 in the anesthetized male condition (AM). Sonograms were analyzed and the following spectral features were calculated automatically by the Mouse Song Analyzer MATLAB code from the sonograms of each of the classified syllable types: Syllable duration, inter-syllable interval (ISI), mean (pitch), minimum, maximum, start, and end frequencies, frequency modulation, spectral purity, amplitude, and bandwidth. Spectral purity was calculated as the instantaneous maximum power at the peak frequency normalized by the instantaneous total power in the spectrum, averaged across the entire syllable; a pure tone has a spectral purity of 1, and white noise approaches 0. In the main text, we only report on five main features (e.g. mean frequency), as more minor features (e.g. end or start mean frequencies) did not reveal new information relative to the main features.

### Syntax/sequence analyses using probabilities

Following a method described in a previous study of ours (Chabout et al., 2015), we used our custom script generated in Microsoft Excel (2013) that detects silences (gap > 250 ms), and letter-coded sequences of syllables and silence (Chabout et al., 2015). These data were used to calculate the “conditional probabilities” of different syllable transition types for each mouse:

(1) $$\begin{array}{l} Probability\left(\begin{array}{l} occurence\;of\;a\;transition\;type\\ \;given\;the\;starting\;syllable \end{array}\right)\\ \;=\frac{\begin{array}{l} Total\;number\;of\;occurences\;of\;a\\ \;transition\;type \end{array}}{\begin{array}{l} Total\;number\;of\;occurences\;of\;all\\ transition\;types\;with\;the\;same\;staring\\ \;syllable\\ \; \end{array}} \end{array}$$

We then averaged the probabilities from all males within a group and contexts, to obtain conditional probabilities for the entire group. We graphed these group-context conditional probabilities into syntax diagrams using Graphviz v2.36 (http://www.graphviz.org/), with nodes designating different syllable categories or silence, and arrows the transitions between the syllables and silence. Arrow thickness in pixel size was made proportional to the conditional probability values. In the diagrams, we only include transitions that were produced by the mice with a probability higher than 0.05 to show the common transitions, and not rare events. The statistical analyses of syntax described below include all transitions recorded, even if rare, such as produced by one animal.

Using these conditional probabilities, we then investigated whether the transition dynamics, as characterized by these transition probabilities, varied significantly between the two genotypes, using two different modes of a novel statistical approach. The first allowed us to test for statistical differences in transition dynamics in the animals from the same genotype between two different contexts. The second allowed us to test the differences in transitions between two independent groups of animals from two different genotypes within the same context. This approach allowed us to test differences in transitions *to* and *from* different syllables, and provided additional insights into differences in individual transition types that made up these sets of probabilities. The procedure comprised two stages. In the first stage, we focused separately on each of the 24 transition types and tested whether the corresponding context-specific or genotype-specific distributions are different. We used non-parametric rank based tests, avoiding restrictive parametric assumptions on the transition probabilities. For the within genotype comparisons between contexts, we used paired sample Wilcoxon signed rank sum tests (WSR). For the between genotype comparisons within the same context, we used two sample Wilcoxon-Mann-Whitney (WMW) tests. In the second stage, we combined the *p*-values returned by these “local” tests to obtain test statistics and *p*-values for testing the differences in the transition probabilities *to* and *from* different syllables as well as for testing the differences in the global dynamics. We used the method of Zaykin et al. (2002), which is robust to the presence of a few outlying local *p*-values. The null distributions of the combined test statistics were determined using a permutation based Monte Carlo method that accounts for the correlation among the local *p*-values. The *p*-values for the combined tests were finally corrected for multiple combined tests corresponding to different syllables. We include in the supplement our R program generated scripts (which we called Syntax Decoder) for the syntax analyses. See Data Sheet 1 in Supplementary Material for additional details.

### O_2_ and CO_2_ respiratory measurements

At ~20 weeks of age Oxygen (O_2_) consumption and carbon dioxide (CO_2_) production were measured in 8 C57 wildtype and 9 *Foxp2-R552H* heterozygous mice individually using CLAMS chambers from Columbus Instruments system (Columbus, OH). Measurements were recorded every 20 min over ~48 h. The respiratory exchange ratio (RER) was calculated as the ratio of CO_2_ production (V_CO2_) to O_2_ consumption (V_O2_) at any given time (Thupari et al., 2002). V_CO2_ is the expired CO_2_ volume at ml/kg·h and V_O2_ is the expired O_2_ volume in the same units.

### Double labeling tracer experiment and Foxp2 immunocytochemistry

After all their recording sessions, six heterozygous and six wildtype males were used to trace the connections between the laryngeal muscles and the primary motor cortex M1 following a procedure described in Arriaga et al. (2012, 2015). We used a recombinant strain of pseudorabies Bartha (PRV-Bartha) expressing enhanced Green Fluorescent Protein (eGFP) under the control of the histomegalovirus immediate-early gene promoter (Smith et al., 2000; Card and Enquist, 2001). Live virus was received from Dr. Lynn Enquist's laboratory at Princeton University at a titer of 9.55 × 10^8^ pfu/ml (Virus center grant #P40RR018604), aliquoted at 4 μl per tube, then stored at −80°C, and thawed immediately before injection. General anesthesia was induced with 1% isoflurane. A midline incision of ~1.5 cm was made under the hyoid bone. The skin, fat tissue and membranes were carefully separated to allow access to the deep muscles. We gently pulled back the sternohyoid muscle to expose the larynx and its muscles. A total of 1 μl was injected into the cricothyroid muscle at a rate of 0.05 μl per min using a Nanofil microsyringe system with a 34-gauge stainless steel needle (World Precision Instrument, Sarasota, FL). After 5 min, the micromanipulator was retracted, and the same injection was repeated for the cricoarythenoid lateralis muscle. Injections were made bilaterally in both muscles. A single puncture point was made for the injection to avoid any leakage outside the muscles and spreading to other tissues.

About 120 h after infection, when the virus is expected to infect 2nd order LMC neurons (Arriaga et al., 2012, 2015), mice were given an overdose of pentobarbital sodium and perfused transcardially with 0.1M PBS followed by 4% paraformaldehyde (PFA) in 0.1M PBS. Brains were removed, post fixed in 4% PFA overnight, then cryoprotected in 30% sucrose in 0.1M PBS until they sank at the bottom of the tube. Brains were then frozen in TissueTek® O.C.T. compound. Forty micrometer coronal sections were cut on a cryostat and put into 0.1M PBS. Forebrain (from +0.50 mm to −0.46 mm) sections were mounted directly on SuperFrost® Plus slides with Vectastain with DAPI (Vector Labs) to observe eGFP expression pattern. Pictures of the slides where taken either with Olympus DVX10 or Olympus BX61 (for high magnification). Then the number of positive layer V neurons in M1 per section was quantified manually and graphed in an Excel (2013) file.

To measure the expression profile of Foxp2 protein in these and adjacent neurons, we unmounted the sections with PRV positive cells in 0.1M PBS and stained them with a FOXP2 antibody (abcam 160046). Sections were washed 3 times in 0.1M PBS, then incubated in 0.1M PBS + 0.3% Triton (X100) + 10% NGS for 1 h at room temperature. Section were incubated overnight at 4°C with anti-FOXP2 at a 1:5000 dilution in 0.1M PBS + 3% BSA + 0.3% Triton (X100) + 10% NGS. After three washes in 0.1M PBS, a fluorescent secondary anti-Rabbit Cy3 anti-body was used at a 1:500 dilution in PBS 1X + 3% BSA + 0.3% Triton (X100) +10% NGS for 1.5 h at room temperature. Washed sections were mounted with DAPI medium (Vectashield) and coverslipped.

### Statistical analysis of acoustic features and repertoire composition

Statistical analyses were conducted using either IBM SPSS Statistic software (v.22.0) or R (R Development Core Team, 2011). Two-way repeated measures ANOVA or MANOVA were used to compare male subject performances across genotypes, stimuli, or across syllable types. For the Repeated-measure ANOVA, when the assumption of sphericity was violated (Mauchly's test) we reported the corrected degrees of freedom using Greenhouse-Geisser correction. *Post-hoc* analyses were performed using WMW tests for independent variables. Student's paired *t*-test comparisons were used for dependent variables when appropriate.

## Results

We compared acoustic features and sequencing of four major USV syllable categories [simple (s), down (d), up (u), and multiple (m) pitch jumps; Figure 1D] from wildtype (*n* = 8) and *Foxp2-R552H* heterozygous (*n* = 10) male littermates in four social contexts: with female urine [UF], live female [LF], anesthetized female [AF], or anesthetized male [AM]. Previously, we found that the B6D2F1/J strain of male mice produce differences in their song repertoires in these four contexts (Chabout et al., 2015), and thus, we thought it prudent to characterize vocal behavior in heterozygous mice in each context separately.

### *Foxp2-R552H* heterozygotes produce normal syllables and at normal rates

**Figure 2:**
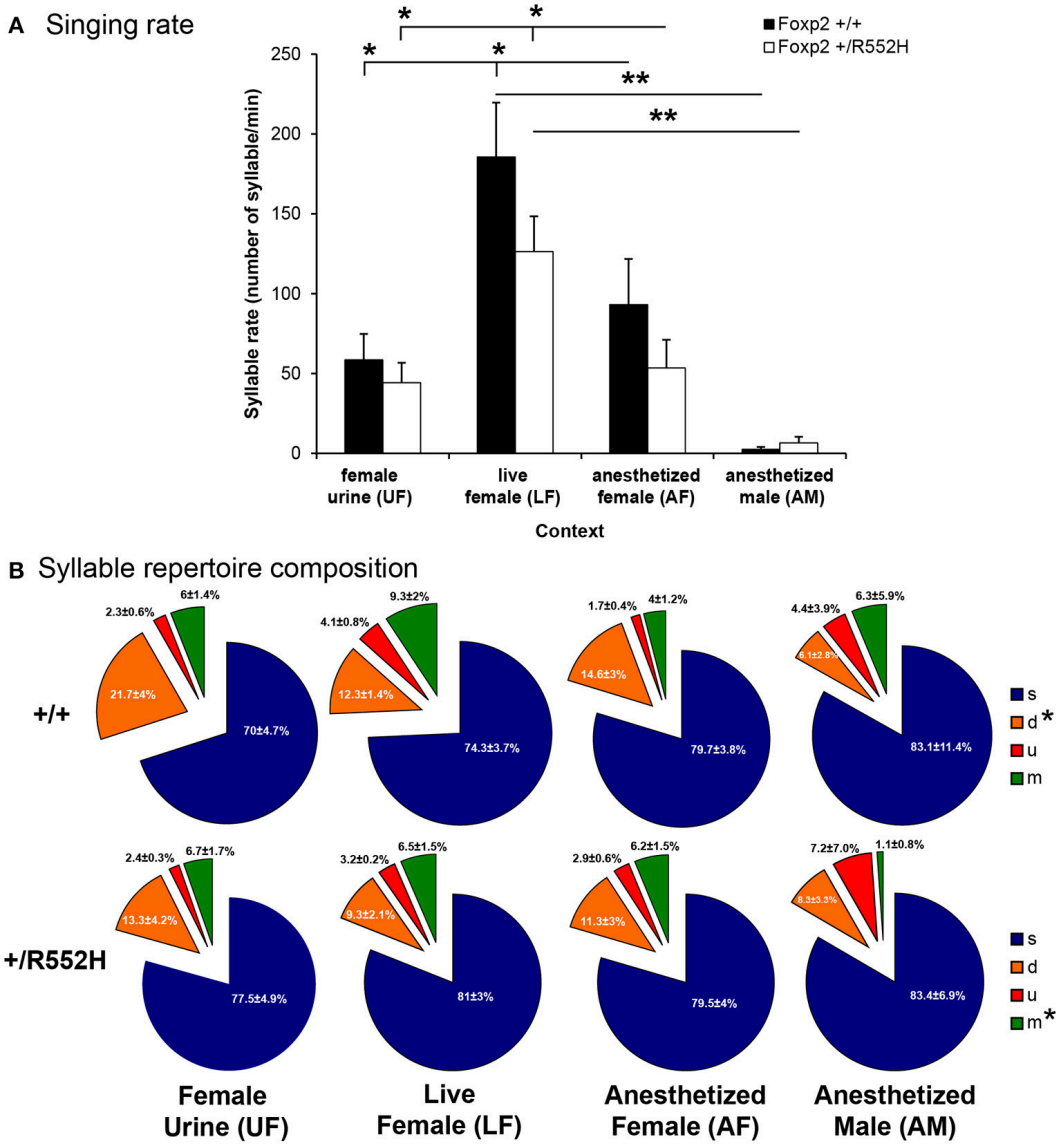
**Syllables production rate and repertoire composition across contexts**. **(A)** Syllable production rate for wildtype (*n* = 8) and *FoxP2-R552H* heterozygous (*n* = 10) mice in each context. Data are presented as mean ± SEM. ^*^p < 0.05, ^**^p < 0.005 for *post-hoc* Student's paired *t*-test after Benjamini-Hochberg correction. **(B)** Repertoire compositions of the four major syllable categories in each context. ^*^p < 0.05 repeated measure ANOVA across contexts for a given syllable type and genotype.

Since the *Foxp2*-*R552H* mutation was backcrossed on a different wildtype background (C57BL6/J, hereafter called C57) than the strain used for our previous study (B6D2F1/J, hereafter called B6; Chabout et al., 2015), we first checked whether the C57 wildtype also showed social context differences. Although we did not find social context differences in acoustic features of C57 adult male USV song syllables (Figure S1), they produced higher rates of syllables in the presence of a live female (LF; Figure 2A [unlike B6 males which had highest rates for fresh female urine UF (Chabout et al., 2015)]. Like B6 males (Chabout et al., 2015), C57 males produced intermediate rates in the presence of an anesthetized female (AF) and very few or no syllables in the presence of an anesthetized male (AM; Figure 2A).

Relative to C57 wildtypes, the *Foxp2* heterozygous C57 male littermates did not differ in any acoustic features measured (Figure S1 white vs. black bars). However, in female-associated contexts (UF, LF, and AF), *Foxp2* heterozygotes had a non-significant trend for lower syllable production rates (Figure 2A), which was related to an interaction with sequence length, described later in this study. These adult findings are consistent with a previous study on pup calls (Gaub et al., 2010), which found no differences in syllable acoustic structure or production rate in *Foxp2-R552H* heterozygotes.

### *Foxp2-R552H* heterozygotes have subtle differences in repertoire composition

Relative to B6 males in our previous study (Chabout et al., 2015), C57 wildtype males in the current study produced fewer differences in syllable repertoire composition across context, where only the down “d” pitch jump syllable type was produced proportionally more in the presence of female-associated stimuli (UF, LF, and AF) compared to anesthetized males (AM; Figure 2B). *Foxp2* heterozygous males lost the “d” syllable context-dependent difference, and also produced proportionally less complex multiple “m” pitch-jump syllables in the anesthetized male context (Figure 2B). Despite these within-genotype effects, differences were not detected when comparing between genotypes. These findings suggest subtle differences in context-dependent syllable repertoire composition in *Foxp2* heterozygotes, which appear to affect production of more complex syllables.

### *Foxp2-R552H* heterozygotes have altered sequence structure

**Figure 3:**
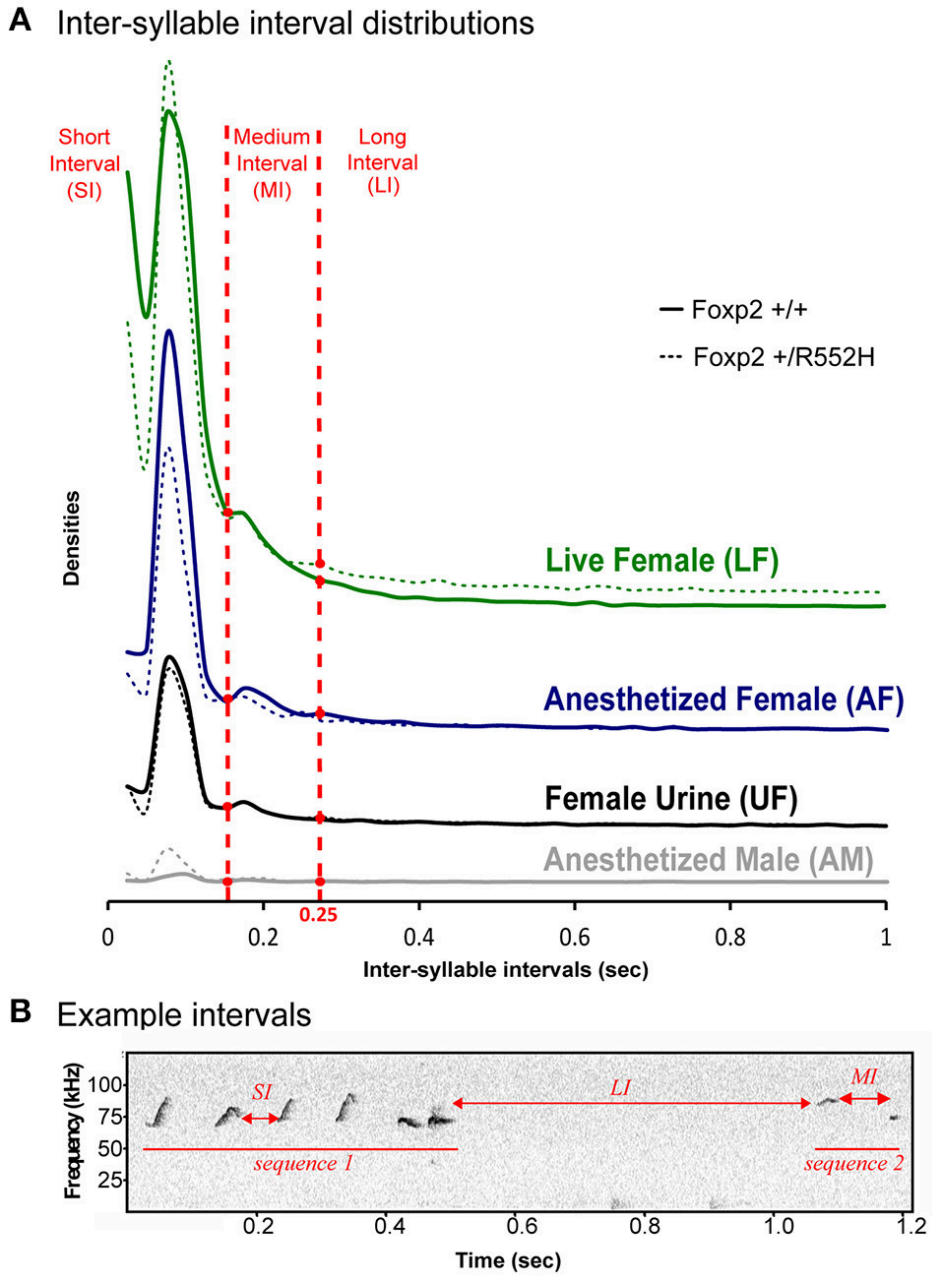
**Temporal organization of sequences in different contexts**. **(A)** Distribution of the inter-syllables intervals, for the four context (colors), defining three types of silent intervals (horizontal red dashed lines) in sequences of syllables for wildtype (*n* = 8) and *FoxP2-R552H* heterozygous (*n* = 10) mice. **(B)** Sonogram showing example syllable sequence intervals of a C57 wildtype male.

To analyze syllable syntax (i.e., sequencing), we used our previous approach of defining a song-bout sequence based on automated quantification of Inter-Syllable Intervals (ISI; Chabout et al., 2015). Similar to B6 males (Chabout et al., 2015), C57 males had several peaks in ISI distribution, with the shortest two [short interval (SI) and medium interval (MI)] corresponding to silences between syllables within a bout, and a longer interval (LI) of 250 ms or more (2 times the *S.D*. of the central peak) corresponding to separating sequences (Figures 3A,B). There were no overt differences in ISI peak timing between wildtypes and *Foxp2* heterozygotes within or across contexts (Figure 3A).

**Figure 4:**
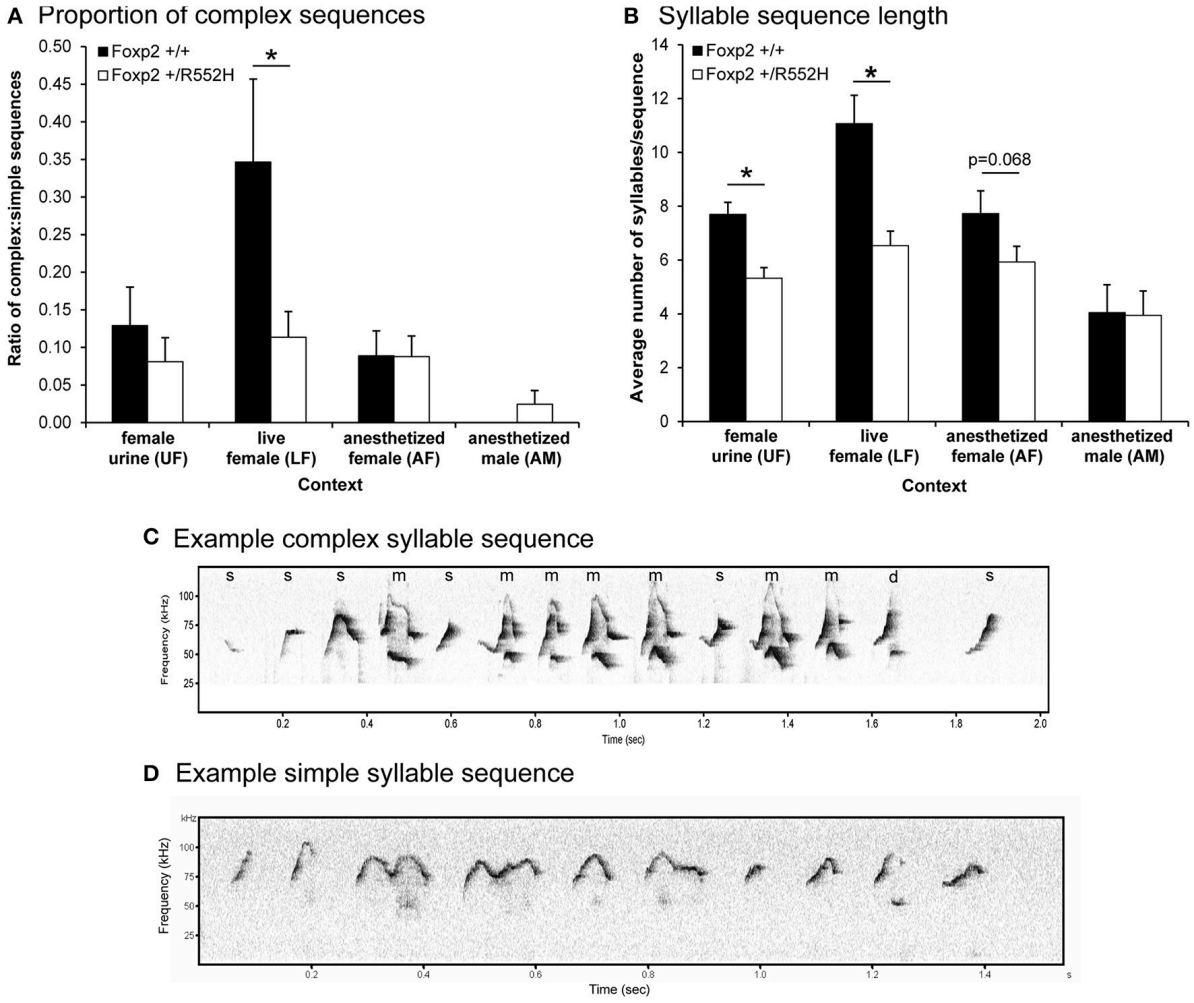
**Sequence measures for each context**. **(A)** Ratio of complex song syllable sequences over simple songs in each context. Graphed are the number of sequences with two or more complex “m” syllables divided by the number of sequences with one or no “m” syllables in each context. Sequences with two syllables or less were not included. **(B)** Lengths of syllable sequences. Graphed are the average number of syllables per sequence, regardless of the total length of the syllables or sequence in seconds. Data are presented as mean ± SEM. ^*^p < 0.05 using Wilcoxon-Mann-Whitney tests for independent samples (*n* = 8 WT; 10 heterozygous). The values for the AF group approached significance. **(C,D)** Example sonograms of longer complex and shorter simple syllable sequence differences between wildtype and *Foxp2-R552H* heterozygous mice, respectively, in the LF context.

We next measured the ratio of complex sequences (containing at least two occurrences of the complex syllable type “m”) vs. simple sequences (containing one or no “m”) in the different contexts, and found that in contrast to B6 males in the female urine context (Chabout et al., 2015), wildtype C57 males produced a >3-fold increase in sequences with complex “m” syllables specifically in the live female context (LF; Figures 4A,C,D). *Foxp2* heterozygotes lost this context-dependent increase (Figures 4A,C,D). We know that females (at least B6) prefer to listen to these more complex pitch jump syllable sequences (Chabout et al., 2015).

**Figure 5:**
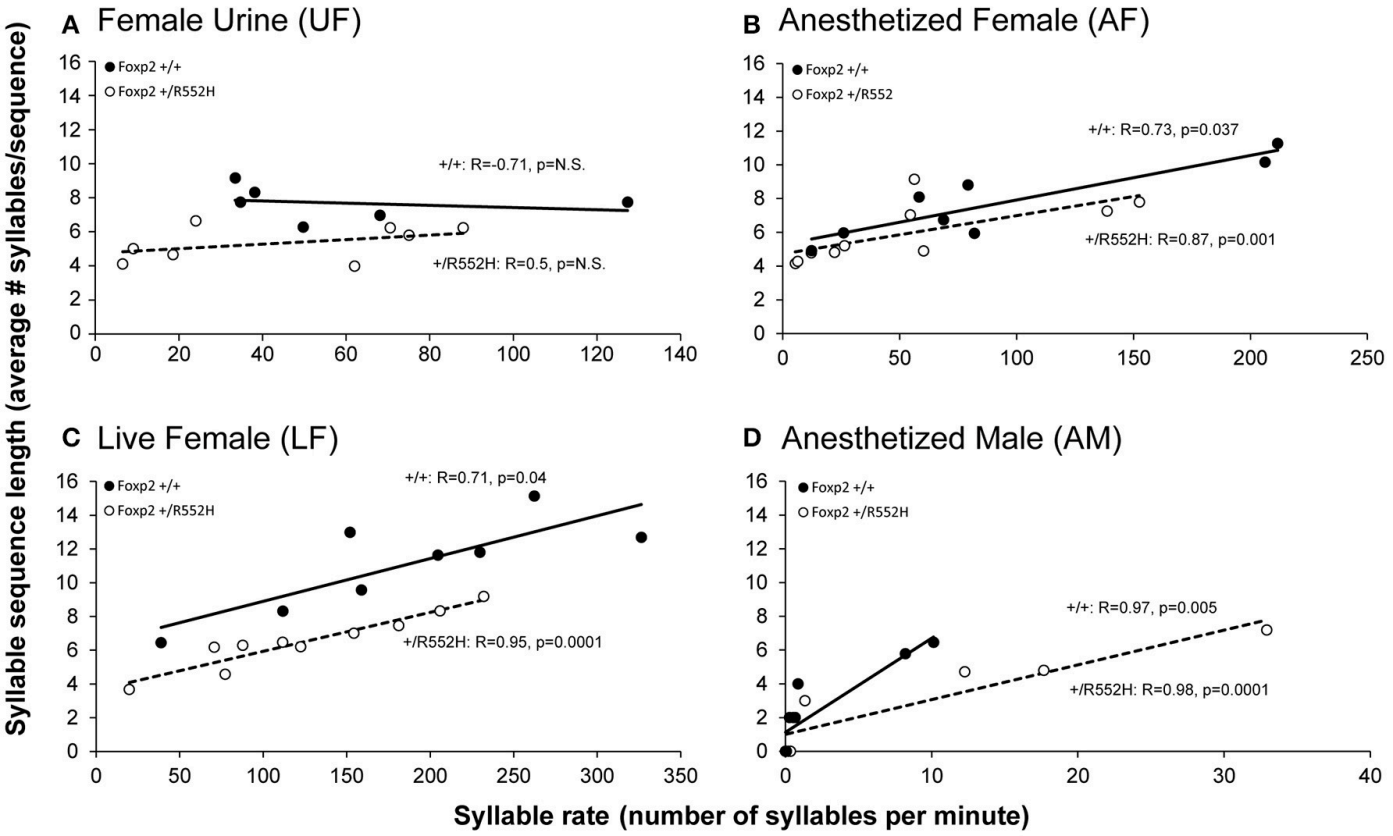
**Correlations between syllable sequence length and syllable rate across context**. Shown are correlations in wildtype (*n* = 8) and *Foxp2-R552H* heterozygous (*n* = 10) mice in the four context: **(A)** Fresh female urine (UF); **(B)** Live female (LF); **(C)** Anesthetized female (AF); and **(D)** Anesthetized male (AM). The x-axes are not drawn to the same scale, since the males produced greater differences in ranges of syllable production rates than sequence lengths (y-axes) across contexts. The correlations in the AM context **(D)** are still significant when removing from the analyses animals that did not sing (0 syllables; +/+*R* = 0.976, *p* = 0.005; +/R552H *R* = 0.988, *p* = 0.0001). Statistics are Spearman's correlation.

*Foxp2* heterozygous males also produced shorter sequences (i.e., a lower number of syllables per sequence) than their wildtype littermates in the female associated contexts (UF, LF, AF; Figure 4B). Additionally, there was a positive correlation between syllable sequence length (Figure 4B) and production rate (Figure 2A) in all contexts involving the presence of another animal (LF, AF, and AM; Figure 5). However, only in the live female context was there a difference in the correlations (slopes) between genotypes, where the heterozygotes produced both proportionally shorter sequences and lower syllable rates (Figure 5C).

**Figure 6:**
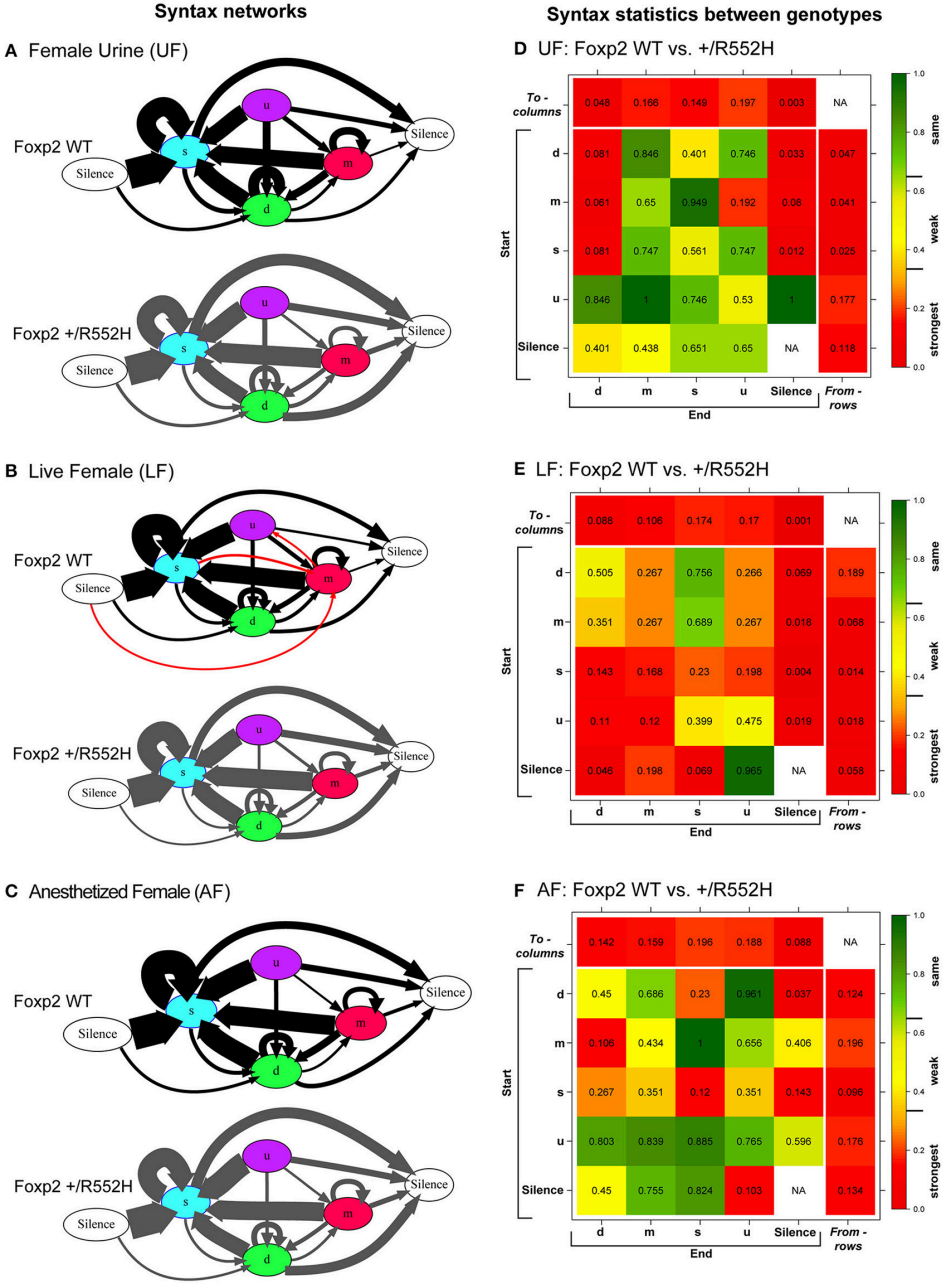
**Syntax analyses**. **(A–C)** Diagrams representing conditional probabilities (for those produced at *p* = 0.05 or greater) of syllable transitions within song sequences in each context and genotype. Arrow thickness is proportional to probability value of going from one syllable type to another (averaged from *n* = 8 WT; 10 heterozygous males). Red colored arrows are transitions produced by wildtype in the LF context that add to increased diversity. **(D–F)** Heat map distributions of the statistical probabilities of differences between wildtype and *Foxp2-R552H* heterozygous mice for each transition type across contexts. For each of the 24 transition types we tested whether the corresponding group-specific distributions are equal between genotype (WMW). Combined *p*-values returned by these “local” WMW tests provide test statistics and *p*-values for testing the differences in the transition probabilities *to* (*columns*) and *from* (*rows*) different syllables. The individual cells within correspond to transitions from (start) a given syllable type to (end) a given syllable type. Figure S3 shows the *to* (*columns*) and *from* (*rows*) *p*-values for multiple tests using Benjamini-Hochberg correction.

The above findings led us to investigate whether there were differences in internal song sequence structure of heterozygous animals. We calculated the conditional probabilities of different transition types (i.e., with fixed starting syllables; Figure S2) and generated graphical syntax diagrams (Chabout et al., 2015) (Figures 6A–C; for common transitions with an occurrence >0.05 probability; red lines in Figure S2). Similar to B6 male mice (Chabout et al., 2015), C57 wildtypes in all contexts typically started a sequence with the “s” syllable type, followed by either looping with the “s” type or transitioning to the “d” and then to other syllable types (Figures 6A–C). At this probability cut-off level, the “s,” “d,” and “m” types were repeated in loops, whereas the “u” type was not. However, instead of producing greater syntax diversity in the female urine context as previously found in B6 males (Chabout et al., 2015), C57 males produced greater syntax diversity in the live female context, also involving transitions with “m” type syllables (Figures 6A–C). The *Foxp2* heterozygotes produced all the same syllable transition types as the C57 wildtype in the urine and anesthetized female contexts, but they did not switch to the more diverse syntax in the live female context (Figure 6B). Instead, syntax of heterozygous animals in the presence of live females was more similar to the socially-reduced contexts (urine only or anesthetized females). There also appeared to be differences in relative proportions of transition types between wildtypes and heterozygotes under different social contexts (Figures 6A–C; differences in syllable transition probabilities, represented by arrow thickness).

To determine whether these syntax findings are statistically different, we could not use our previous approach (Chabout et al., 2015) as it was only sufficient for comparing differences within the same animals from one condition (e.g., context) to another. Thus, we developed a new approach based on Markov chain frameworks, Wilcoxon-Mann-Whitney rank sum tests, and Monte Carlo permutations, to test whether the syllable transition dynamics varied significantly between two groups of animals (two sample test), i.e., wildtypes and heterozygotes, within contexts, as well as between different contexts (paired test) within genotypes (see Section Methods and Data Sheet 1 in Supplementary Material). We tested for statistical differences at three levels: globally for the entire syntax network; for all transitions *to* and *from* a particular syllable type; and for individual transitions between two specific syllable types. In the *to syllable* case, we asked: when starting with different syllable types (“d,” “m,” “s,” “u,” or silence), do the probabilities of transitioning to a particular specified syllable type (say “d”) differ between the two groups of animals? In the *from syllable* case, we asked: when starting with a particular specified syllable type (say “m”), do the probabilities of transitioning to different syllables (“d,” “m,” “s,” “u,” and silence) differ between the two groups? These analyses included all transitions, whether they were produced at < 0.05 occurrence.

**Figure 7:**
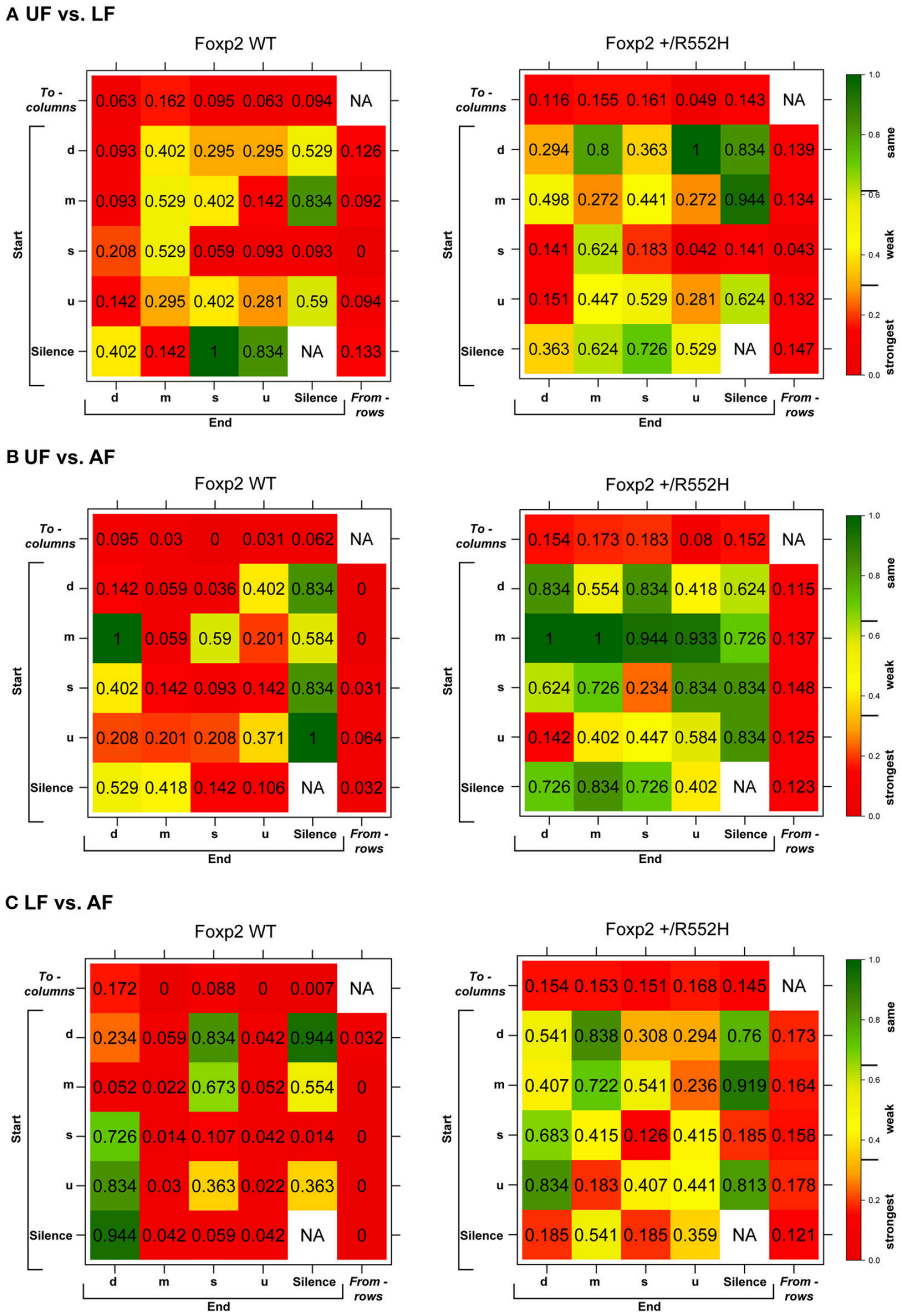
**Syntax comparisons across contexts**. **(A–C)** Heat maps distributions of the statistical probabilities of differences between **(A)** UF and LF, **(B)** UF and AF, **(C)** LF and AF for wildtypes (WT; left columns) and *Foxp2-R552H/*+ heterozygotes (right columns). For each of the 24 transition types, we tested whether the corresponding group-specific distributions are equal between contexts (See Data Sheet 1 in Supplementary Material). Combined *p*-values returned by these “local” tests provide statistics and *p*-values for testing the differences in the transition probabilities *to* (*columns*) and *from* (*rows*) different syllables. The individual cells within correspond to transitions from (start) of a given syllable type to (end) of a given syllable type.

In the pairwise analyses with genotypes fixed, consistent with the syntax graphs, C57 wildtypes had global statistically significant syntax differences between contexts (e.g., AF vs. UF and AF vs. FE), whereas *Foxp2* heterozygotes did not (Table 1A). When examining transitions *to* (top row) and *from* (last column) different syllable types, relative to C57 wildtypes, *Foxp2* heterozygotes had weaker differences in transition probabilities (greener colors/higher *p*-values), particularly in the anesthetized female context (Figure 7). These global *to* and *from* transition differences were due to differences in specific syllable transition types in the heterozygotes across contexts compared to wild type (Figure 7; greener colors in inner cells of heatmaps for heterozygotes).

**Table 1:** **Comparison of syntax dynamics: (A) Table of global *p*-values across contexts; (B) Table of global *p*-values between genotypes**.

**A: GLOBAL** ***P*****-VALUES BETWEEN CONTEXTS**		
**Condition**	**Wild-type**	***Foxp2-R552H***
UF vs. AF	<0.0001	0.5243
UF vs. LF	0.094	0.2908
LF vs. AF	<0.0001	0.4677
**B: GLOBAL** ***P*****-VALUES ACROSS GENOTYPES**		
**Condition**	***p*****-values**	
UF	0.052	
AF	0.2913	
LF	0.0255	

In the two sample analyses directly comparing genotypes, consistent with the pairwise analyses, the heterozygotes had global statistically significant syntax differences with wildtype, in the urine and live female contexts (Table 1B; Figures 6D,E). This was in part due to relatively stronger differences in transition probabilities *to* (top row) silence in the urine and live female contexts (Figures 6D,E); these relative differences survive a Benjamini and Hochberg false-discovery-rate correction for multiple testing (Table S1; Figure S3). When examining the specific transitions that contributed to these differences (inner cells of the heat maps in Figures 6D–F; Figure S2 for direction of the changes), the strongest differences between heterozygous and wildtype animals were in the transitions from all syllables to silence in the live female context (Figure 6F and Figure S2B), from all syllables except “u” to silence in the female urine context (Figure 6D and Figure S2A), and mainly from “d” to silence in the anesthetized female context (Figure 6E and Figure S2C). These differences can be explained by two types of global transition changes: (1) heterozygous mice producing more transitions to silence in all contexts (thicker arrows for heterozygotes in Figures 6A–C), consistent with heterozygotes producing shorter sequences; and (2) heterozygous mice producing decreased transitions from other syllables to “d” (Figures 6A,D) and from “m” syllables to other syllables (Figures 6C,F), consistent with the analyses within genotype.

Taken together, the above findings indicate that compared to wildtypes, heterozygous males produced shorter sequences in most contexts (due mainly to transitioning to silence more often from specific syllable types), had reduced internal sequencing with more acoustically complex syllable types, and did not increase syntax diversity with live females.

### Most differences in *Foxp2-R552H* heterozygotes are stable across season and experimental paradigm

The strain and genotype differences in the results above, subtle in some cases and large in others, along with the variable conclusions in different studies on pup calls (Shu et al., 2005; French et al., 2007; Groszer et al., 2008; Gaub et al., 2010; French and Fisher, 2014) led us to seek replication of the key findings in an independent set of 31 animals (16 heterozygous and 15 wildtype males) and at a different time of the year (Fall/October instead of Spring/March). The only other difference from the first set of experiments was that we performed analyses across contexts in a sequential fashion (UF, AF, and FE order) instead of a randomized design (see Section Methods).

Overall, the results were consistent with the first set of experiments: no significant differences between genotypes in the acoustic features measured (Figure S4); switching to a higher ratio of complex-vs.-simple sequences in wildtype and absence of such switching in heterozygotes in the live female context (Figure S5A); and shorter sequences in heterozygotes (Figure S5B). However, we noted some differences compared to the first experiments: a higher rate of singing in the anesthetized instead of live female context for both genotypes (Figure S5C vs. Figure 2); a higher rate of singing for the heterozygous animals in the urine context (Figure S5C), even though they had shorter sequences (Figure S5B); increased use of “m” syllables in the repertoires of both genotypes (Figure S5D vs. Figure 4), and differences in the exact transitions that differed between heterozygotes and wildtypes in each context (Figure 6 vs. Figure S6). Such variability between experiments could be due either to the random vs. sequential experimental design, a motivation to sing more complex courtship songs in the fall vs. the spring, or some other unmeasured variable. The findings, however, remain consistent with our main conclusions that *FoxP2* heterozygotes produce less complex and shorter syllable sequences relative to wildtypes under the same conditions.

### Altered sequencing of *Foxp2-R552H* heterozygotes is not due to differences in respiration

The production of shorter USV sequences in heterozygotes led us to wonder if this could be due to shortness of breath compared to wildtype. We examined the consumption/production rates of oxygen and carbon dioxide (V_O2_/V_CO2_) in all mice in a 48-h period using isolated CLAMS (Comprehensive Lab Animal Monitoring System) chambers. Although, we found some large differences among some animals, there was there was no significant difference between genotypes [repeated measures ANOVA: V_O2_: *F*_(1, 13)_ = 1.279, *p* = 0.27; V_CO2_: *F*_(1, 13)_ = 0.544, *p* = 0.47; RER: *F*_(1, 13)_ = 3.83, *p* = 0.072; Figure S7]. These findings indicate no detectable deficits in respiration in heterozygotes that could explain their production of shorter sequences.

### Position of laryngeal motor cortex neurons is shifted in *Foxp2-R552H* heterozygotes

It has been proposed that human *FOXP2* may contribute to speech acquisition and production through effects on vocal motor pathways of the cortex and basal ganglia, as the human LMC region and parts of the anterior striatum both show altered activation in human KE family *FOXP2* heterozygotes during speech/language-related tasks (Liégeois et al., 2003). The recently discovered mouse rudimentary LMC region that projects to the anterior striatum and to nucleus ambiguous (Amb) brainstem vocal motor neurons (Figure 1A; Arriaga et al., 2012) is within the same coordinate region where *Foxp2* is conspicuously expressed in layer-5 neurons of M1 compared to other parts of M1 (Hisaoka et al., 2010; Pfenning et al., 2014). This prompted us to ask whether these LMC layer-5 neurons have any change in connectivity or other properties in heterozygous mice.

**Figure 8:**
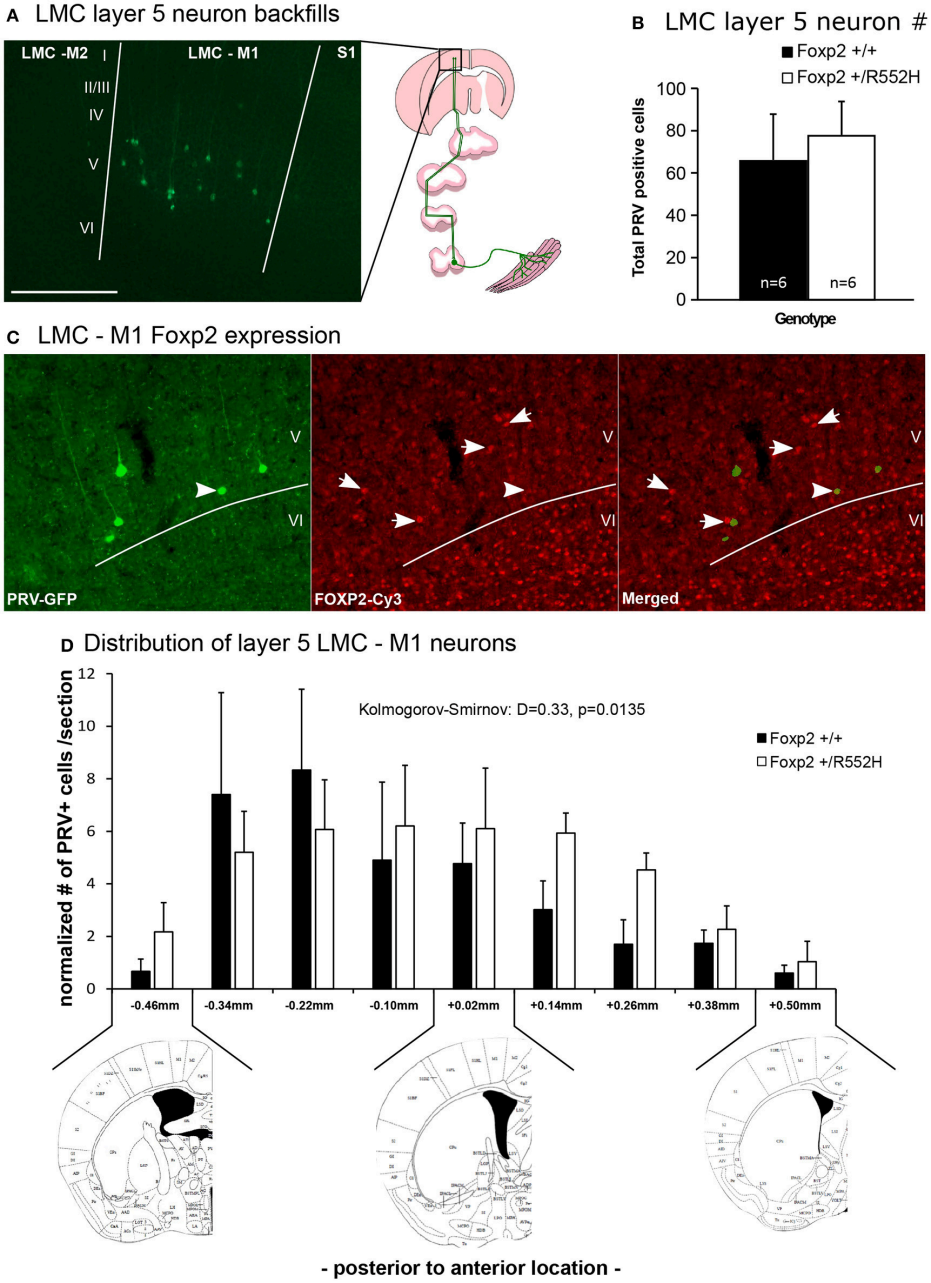
**Retrograde tracing of the laryngeal motor cortex neurons**. **(A)** Example of GFP-labeled (green) layer 5 neurons in mouse LMC-M1 from a pseudorabies virus (PRV) unilateral injection in the cricothyroid and cricoarytenoid lateralis larynx muscles (diagram to right) of a C57 male mouse. Roman numbers correspond to different layers of the cortex as determined in DAPI counterstaining. Section is coronal, contralateral hemisphere to muscle injection. Scale bar, 500 μm. Left image schematic from (Arriaga et al., 2015). **(B)** Total number of PRV-GFP positive cells labeled from all rostral to caudal coronal sections processed in wildtype and *Foxp2-R552H* heterozygous mice. No significant difference was found (*p* = 0.42; Wilcoxon-Mann-Whitney tests for independent samples). **(C)** Example double labeling of GFP-backfilled (green) LMC layer 5 neurons and Foxp2 protein expression (red Cy3). Layer 6, as known (Hisaoka et al., 2010), has the highest numbers of neurons with Foxp2 expression, followed by layer 5 in this particular region of the cortex. Arrow, example doubled labeled cell with intermediate levels of Foxp2 expression; arrowhead, example non-backfilled layer 5 cell with high Foxp2 expression. **(D)** Distribution, section-by-section, of the PRV positive cells in both genotypes. Data are presented as mean ± SEM normalized per number of section counted for wildtype and *Foxp2-R552H* heterozygous mice. Kolmogorov-Smirnov test was used to assess the difference between the two distributions (*n* = 6 males per genotype). Anatomical coronal diagrams below the graph show representative locations with coordinates relative to Bregma indicated; images used from The Mouse Brain in Stereotaxic Coordinates, Paxinos G. and Franklin K. B. J. with permission.

Using our previous approach (Arriaga et al., 2012, 2015), we injected laryngeal muscles with a pseudorabies virus that travels retrogradely and transynaptically through functional synapses, and confirmed the presence of M1 LMC layer-5 neurons in C57 male mice (Figure 8A). Double-labeling experiments confirmed that these cells were located in the same region of M1 that has *Foxp2*-expressing layer-5 neurons, but that specific laryngeal connected layer-5 neurons expressed less *Foxp2* (Figure 8B); this difference of less Foxp2 expression could be due to real differences in *Foxp2* expression in laryngeal connected layer-5 neurons or toxicity to the neurons from the pseudorabies virus. The *Foxp2* heterozygotes had these same laryngeal connected layer-5 cells, with no significant difference in the total number of labeled cells (Figure 8C). However, heterozygous mice showed a significant posterior shift and a more shallow peak in the distribution of LMC layer-5 neurons compared to wildtypes, resulting in the heterozygous LMC layer-5 neurons being more spread out in the cortex (Figure 8D). We therefore conclude that the heterozygous *Foxp2* mutation did not change the presence, number, or gross connectivity of these laryngeal premotor neurons, but did alter their relative localization in the cortex. Future studies will be required to determine if there is a causal relationship between the change in distribution of these cells and the alterations in USV sequencing in the heterozygous animals.

## Discussion

Mice do not have the complex vocal learning behavior of humans and song-learning birds (Kikusui et al., 2011; Arriaga et al., 2012; Hammerschmidt et al., 2012, 2015; Arriaga and Jarvis, 2013; Mahrt et al., 2013; Portfors and Perkel, 2014). Nonetheless, we find that the same *FoxP2* mutation in mice and in humans leads to overlapping effects on sequencing of vocalizations. In particular, against a background of preserved syllable acoustic structure, we see reductions in the length and complexity of syllable sequences. Moreover, in both mice and humans carrying the KE mutation, the effects become more profound as the expected sequence becomes more complex. In humans the deficits are manifested more when heterozygous individuals are asked to produce words or non-word vocalizations with more complex sequences of syllables/phonemes, whereas they more easily produce words with just two syllables or less complex sequences of similar syllable types (Hurst et al., 1990; Watkins et al., 2002a). In mice, the analogous effects occur in heterozygotes in a context where wildtype animals normally produce longer and more complex syllable sequences.

The altered sequencing in heterozygous mice may be more subtle than in humans, as heterozygous mice can still sequence many simple syllables together (albeit shorter sequences). The more subtle effect in mice is consistent with the continuum hypothesis of vocal learning or vocal plasticity (Petkov and Jarvis, 2012; Arriaga and Jarvis, 2013), where instead of being completely absent in so-called vocal non-learning species, mice (Arriaga et al., 2012) and some vocal non-learning birds (Liu et al., 2013) have rudimentary behavior and neural circuitry that is present in the more advanced vocal learners like humans and song-learning birds.

Crucially, the tools and ideas we borrowed from the songbird field, novel ones we developed here, and analyses of adult mice in different social contexts have revealed differences in heterozygous animals that were either missed in past studies or not considered. For example, we considered social context as a possible variable that might impact vocal plasticity (Jarvis et al., 1998; Chabout et al., 2015). If we had only used female urine or an anesthetized female to stimulate male USV songs, we would have missed some of the larger effects on syllable sequencing, besides sequence length, produced by heterozygous males in the presence of awake females. Future studies will be necessary to determine whether C57 females, like B6 females, prefer the more complex syllable sequences, and if so, why B6 males produce them more often in the presence of female urine (Chabout et al., 2015). If C57 females find more complex sequences more attractive, then a prediction would be that they would find heterozygous *Foxp2* male songs less attractive [although in our past study a minority of 1–2 females found simpler songs more attractive (Chabout et al., 2015)].

Females also produce USV syllables similar to males, but not as often, and especially not in the presence of vocalizing males (Neunuebel et al., 2015). We do not believe that the increased sequence diversity in the live female context with C57 male mice is due to females vocalizing with the males, as we did not find an appreciable overlap of two animals vocalizing in the sonograms and we did not find the increased sequence diversity when heterozygous males were housed with wildtype females. Future studies would benefit from using triangulation of multiple microphones as recently done for mice housed in small groups (Neunuebel et al., 2015), to determine the USV properties of heterozygous *Foxp2* females in different social contexts.

Most prior studies of vocal behavior in mice with various *Foxp2* disruptions have been largely limited to analyzing pup isolation calls (Fujita et al., 2008; Groszer et al., 2008; Gaub et al., 2010; French and Fisher, 2014). Although some early reports using either *Foxp2* knockouts (Shu et al., 2005) or *Foxp2*-*R552H* heterozygous (Fujita et al., 2008) mice concluded that pups with heterozygous *Foxp2* disruptions display decreased isolation call rates, these initial claims have not been replicated by independent in-depth analyses of pup calls (Gaub et al., 2010). The later study also did not find consistent significant effects on the acoustic measures studied. Although young mouse pups are able to produce complex syllables, the complexity of bouts of vocal sequences increases as the pups age, with a greater tendency to switch between syllable types (Grimsley et al., 2011). Furthermore, while isolation calls may be informative readouts of arousal states and/or motor function, they do not necessarily translate to socially motivated communication. In the present study we went beyond pup calls and acoustic structure of individual syllables to discover changes in syllable sequences in adult heterozygous *Foxp2-R552H* males, in multiple contexts. The acoustic structure results in adults of our study are consistent with the lack of differences in heterozygous *Foxp2-R552H* pups (Gaub et al., 2010).

Two studies conducted in parallel with ours examined whether adult heterozygous mice with *Foxp2* disruptions display vocalization differences (Castellucci et al., 2016; Gaub et al., 2016). Gaub et al. (2016) examined different arousal and emotional contexts in the same *Foxp2-R552H* founder line, but backcrossed to a different strain (C3H/HenNHsd, rather than C57Bl6). Consistent with the present study, all syllable types that were found in wildtype animals also occurred in heterozygotes, with largely similar properties. However, they reported some subtle effects among two contexts (water vs. female urine), including where heterozygous animals had a longer latency to start their first syllable, a longer syllable duration, increased rate of several complex pitch jump syllable types, and louder USVs at higher minimum frequencies with increased overtones, as compared to wildtype littermates. We saw a trend of increased volume in our analyses (not reaching statistical significance); thus this could represent a difference in context and/or strain background among studies. It is not known if heterozygous humans in the KE family produce louder vocalizations in more emotional contexts. Some of the differences that Gaub et al. (2016) reported in the proportion of complex syllables produced by mice in different contexts were those that varied between our two replicate experiments and thus could be influenced by other variables interacting with the mutation. Of note, the Gaub et al. (2016) study did not analyze sequencing properties of USVs.

Castellucci et al. (2016) focused on song development in mice with a heterozygous knockout of *Foxp2*, on the same strain background (C57Bl/6J) as that used in our study. They used live females to stimulate male song, and found that as wildtype juvenile mice got older they produced a higher proportion of what the authors call “long syllables,” which is similar to our complex multiple “m” pitch jump syllables. They found that heterozygous *Foxp2* knockouts had a much more limited increase of long syllables. Consistent with our findings, heterozygous *Foxp2* knockout mice had normal acoustic structure (duration and frequency modulation), shorter syllable sequences, and decreased transition probabilities to the long (our “m” type) syllables, using similar methods based on our previous approach for quantifying sequences (Chabout et al., 2015). Differences with Castellucci et al. (2016) are: we examined multiple contexts and revealed a relationship with social context (that is, complexity differences are mostly restricted to the context with live females); we have a more advanced statistical tool that detected the specific syllable syntax differences; and we examined the brain, revealing a change in the LMC layer-5 neurons in *Foxp2* heterozygotes. Moreover, our study targeted a mutation that directly matched that found in the most well-studied case of human *FOXP2* disruption (the KE family), while Castellucci et al. (2016) employed a standard heterozygous knockout of the gene. The combined findings support the view that *Foxp2* disruptions impact the more complex sequences of vocalizations as the mice mature, in specific social contexts where such sequences are preferred, with a potential neural substrate in the cortex.

Our findings in heterozygous mice show some interesting differences from prior studies in songbirds. In zebra finches, local viral-based *FoxP2* knockdown in the Area X striatal song nucleus during the vocal learning critical period disrupted vocal imitation of the tutor's song (Haesler et al., 2007); the acquired acoustic structure and the duration of song syllables were abnormally variable, whereas the sequencing of the syllables was less affected. *FoxP2* knockdown in adult Area X abolished context-dependent changes in pitch variability, a feature controlled by Area X, while the length of the motifs and the number of introductory notes did not change (Murugan et al., 2013). *FoxP2* levels in Area X show significant variation in singing-driven gene expression levels in different social contexts (Teramitsu and White, 2006), and developmental and seasonal variation during vocal learning periods (Haesler et al., 2004). Vocal behavioral differences in mice vs. songbirds with FoxP2 manipulations could be due to differences between avian and mammalian brains, or between vocal learners and vocal non-learners. Alternatively, the type of genetic manipulation and its location in the brain could make a difference. In the KE family and mice with the matching mutation, the crucial genetic disruption is present in all cells of the body and brain, throughout the entire life of the individual, such that there is a half-dosage of the functional protein in all the cells that normally express it. In the avian studies, the protein product level was reduced by reducing its RNA translation, and in a localized portion of the brain, lateral Area X. The main downstream output of the lateral portion of Area X is to the RA song nucleus analogous to the layer-5-like LMC neurons (Kubikova et al., 2007; Pfenning et al., 2014), which in songbirds mainly controls the acoustic structure of the vocalizations (Hahnloser et al., 2002; Fee et al., 2004). If *FoxP2* were to be manipulated in medial Area X or other parts of the avian vocal learning circuit, then sequencing deficits might be predicted (Hahnloser et al., 2002).

Which brain circuits mediate the effects on vocalization sequences of a *Foxp2* disruption? Our findings of a shift in the position of the LMC layer-5 neurons adds to the candidate regions, and is the first finding that we are aware of in a non-human mammal showing an alteration in a vocalization-related circuit by a *Foxp2* disruption. Furthermore, a recent study showed that when *Foxp1* (a close transcription factor paralogue of *Foxp2*) is deleted in spinal cord motor neuron progenitors, the neurons are shifted to a more medial location (Hinckley et al., 2015). Future studies would need to investigate if the LMC layer-5 neuron shift is causally related to the alterations in vocal sequencing. One would also need to determine if other motor cortex layer-5 neurons are shifted. An alternative or possibly complementary explanation is that the shifted LMC M1 neurons impact the anterior striatal regions they project to (Arriaga et al., 2012). Prior studies on humans and mice with *FOXP2*/*Foxp2* mutations uncovered structural and functional effects on the striatum (Watkins et al., 2002b; Groszer et al., 2008; Vernes et al., 2011; French et al., 2012; French and Fisher, 2014). Studies in humans indicate that cortico-basal ganglia circuits are involved in combining isolated movements into precise and robust sequences targeted to achieve a particular action (Tanji, 2001; Jin and Costa, 2015), and that parts of the anterior striatum are involved in adult vocal learning (Simmonds et al., 2014). The striatum expresses the highest levels of Foxp2 within the forebrain (Haesler et al., 2004; Teramitsu et al., 2004). Perhaps the heterozygous inactivation preferentially impacts an anterior part of the striatum and its LMC input to affect vocal sequencing more than other behaviors. Thus, our work generates specific testable hypotheses for future studies.

When testing such hypotheses, our statistical tools for syllable sequence analyses will be useful (Chabout et al., 2015 and this study). The diverse syllable variability and sequencing in mouse songs have been difficult to quantify. We believe that the novel statistical techniques that we developed in Chabout et al. (2015, in press) and in this paper provide powerful computational tools to analyze their syntax to discover subtle to strong differences between genotypes and social contexts. Using these tools and knowledge from prior experience with songbirds, our findings indicate that mouse USVs are not as stereotyped in sequence as the songs of the commonly studied zebra finch are among songbirds. Therefore, more sophisticated computational tools are necessary to analyze mouse USVs.

In conclusion, a well-studied heterozygous mutation involved in a human speech deficit neither impedes USV production nor affects syllable acoustic features in adult mice. However, advanced statistical tools developed in this paper revealed that it does alter the dynamic organization of syllables in song sequences. This approach should be useful to more fully exploit the mouse vocal communication system for providing insights into the contributions of *FOXP2* and other genes to spoken-language functions in humans.

## Author contributions

JC conducted research, performed analyses, and wrote the paper; AS and DD developed statistical tools and performed analyses; SP and TR conducted research; SF and EJ co-supervised the study and co-wrote the paper.

### Conflict of interest statement

The authors declare that the research was conducted in the absence of any commercial or financial relationships that could be construed as a potential conflict of interest.

## Supplementary material

Figure S1 **Acoustic features measured in each context**. Acoustic features (rows of graphs) were measured from USV vocalizations in the following context: **(A)** Female urine (UF); **(B)** Live female (LF); **(C)** Anesthetized female (AF); **(D)** and Anesthetized male (AM). Data are presented as mean ± SEM. ^*^*p* < 0.05 using Wilcoxon-Mann-Whitney tests for independent samples (*n* = 8 WT; 10 heterozygous males).

Figure S2 **Conditional probabilities for each transition type across context. (A)** UF; **(B)** LF; **(C)** AF contexts for wildtypes (*n* = 8) and *Foxp2-R552H* heterozygous (*n* = 10) mice. Red dashed lines indicate the 0.05 threshold for values used to generate the syntax graphs. For statistical comparisons between genotype for each transition type see values in Figures 6D–F.

Figure S3 **Statistical analyses of syntax with Benjamini-Hochberg correction**. Shown are the Benjamini-Hochberg analyses of the data in Figures 6D–F.

Figure S4 **Acoustic features measured in each context replicate experiment**. Data are presented as mean ± SEM. ^*^*p* < 0.05 using Wilcoxon-Mann-Whitney tests for independent samples (*n* = 15 WT; 16 heterozygous males). Explanation for other items is the same as Figure S1.

Figure S5 **Syllable sequence and syllable repertoire measures replicate experiment. (A)** Ratio of complex song syllable sequences over simple songs in each context. **(B)** Syllable sequences lengths. **(C)** Syllable production rate. **(D)** Repertoire compositions of the four major syllable categories. Data are presented as mean ± SEM. ^*^*p* < 0.05 using Wilcoxon-Mann-Whitney tests for independent samples (*n* = 15 WT; 16 heterozygous males). Explanations for other items are the same as Figures 2A,B, 5C,D.

Figure S6 **Syntax analyses in the replicate experiment**. Explanation the same as Figure 6 (*n* = 15 WT; 16 heterozygous males).

Figure S7 **Respiratory analyses. (A)** V_O2_ readings. **(B)** V_CO2_ readings. **(C)** RER = V_O2_:V_CO2_ respiratory ratio. There are no statistical differences between respiration (y axes) between wildtype (*n* = 8) and *Foxp2-R552H* heterozygous (*n* = 9) males in the curves. Measurements were recorded every 20 min over a ~48 h period (x-axes) in CLAMS chambers.

Table S1 **Raw *p*-values of the difference between wildtype and *Foxp2-R552H*/+ mutant mice for each transition type conditional probability (rows to columns) across contexts using Benjamini-Hochberg (FDR) or Bonferroni (FWER) correction**. Red, significant at *p* < 0.05.

Supplementary File **Syntax decoder R program**. This folder that contains the R programs used in the syntax analysis. We have named the collection of programs “Syntax Decoder.” Tables of transitions syllable frequencies are required to be fed to the codes. To help the user understand the data structure, we have included two such example files (containing tables of transitions for LF and AF contexts), which can be used to understand the data structures and to try out the codes.
