# Supplementary figures and images for: A Foxp2 Mutation Implicated in Human Speech Deficits Alters Sequencing of Ultrasonic Vocalizations in Adult Male Mice
Source: Front Behav Neurosci. 2016 Oct 20;10:197. doi: 10.3389/fnbeh.2016.00197 (PMC5071336; doi:10.3389/fnbeh.2016.00197)

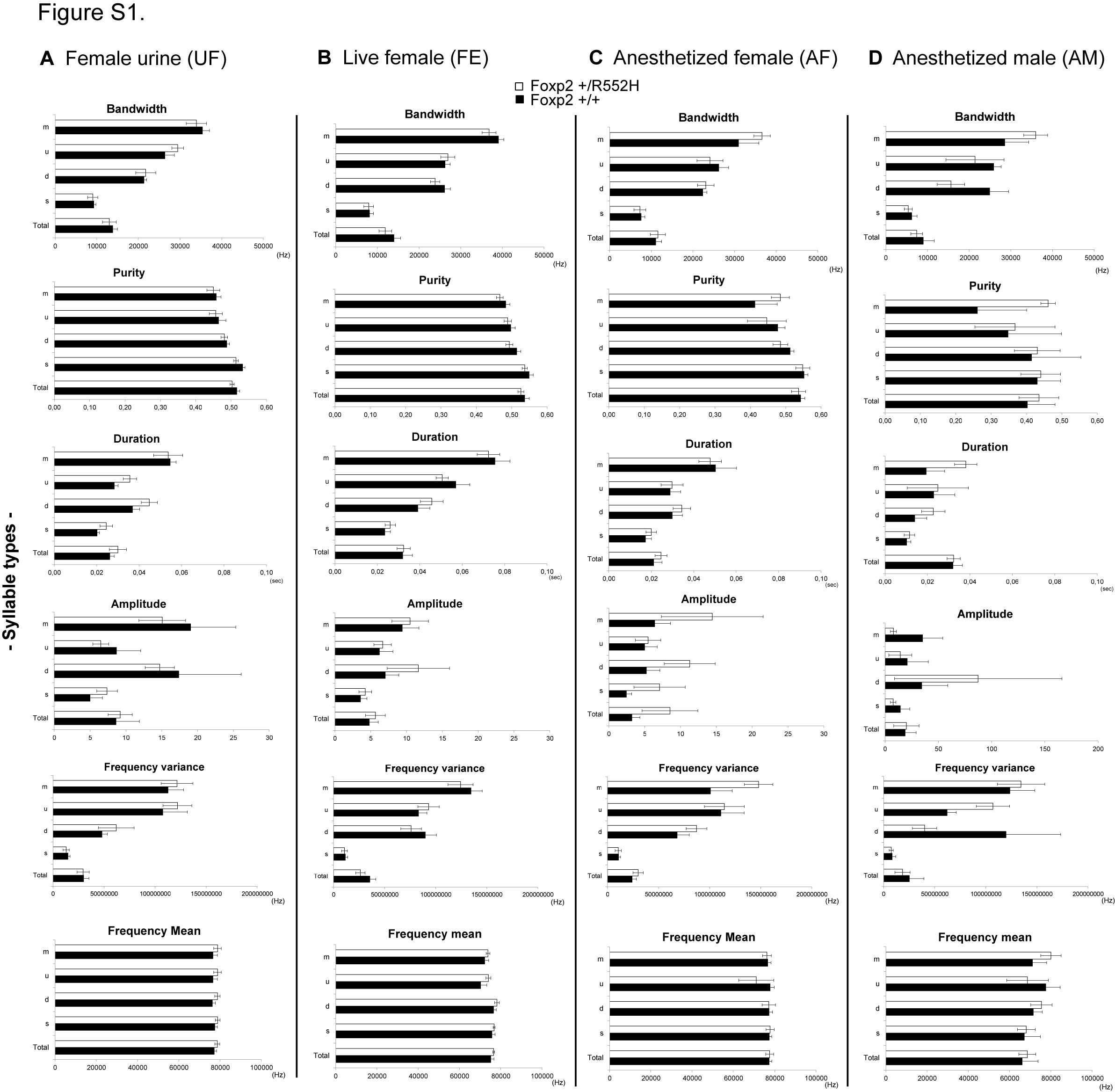

Supplement: Figure S1 — Acoustic features measured in each context. Acoustic features (rows of graphs) were measured from USV vocalizations in the following context: (A) Female urine (UF); (B) Live female (LF); (C) Anesthetized female (AF); (D) and Anesthetized male (AM). Data are presented as mean ± SEM. *p < 0.05 using Wilcoxon-Mann-Whitney tests for independent samples (n = 8 WT; 10 heterozygous males). [file Image1.JPEG]

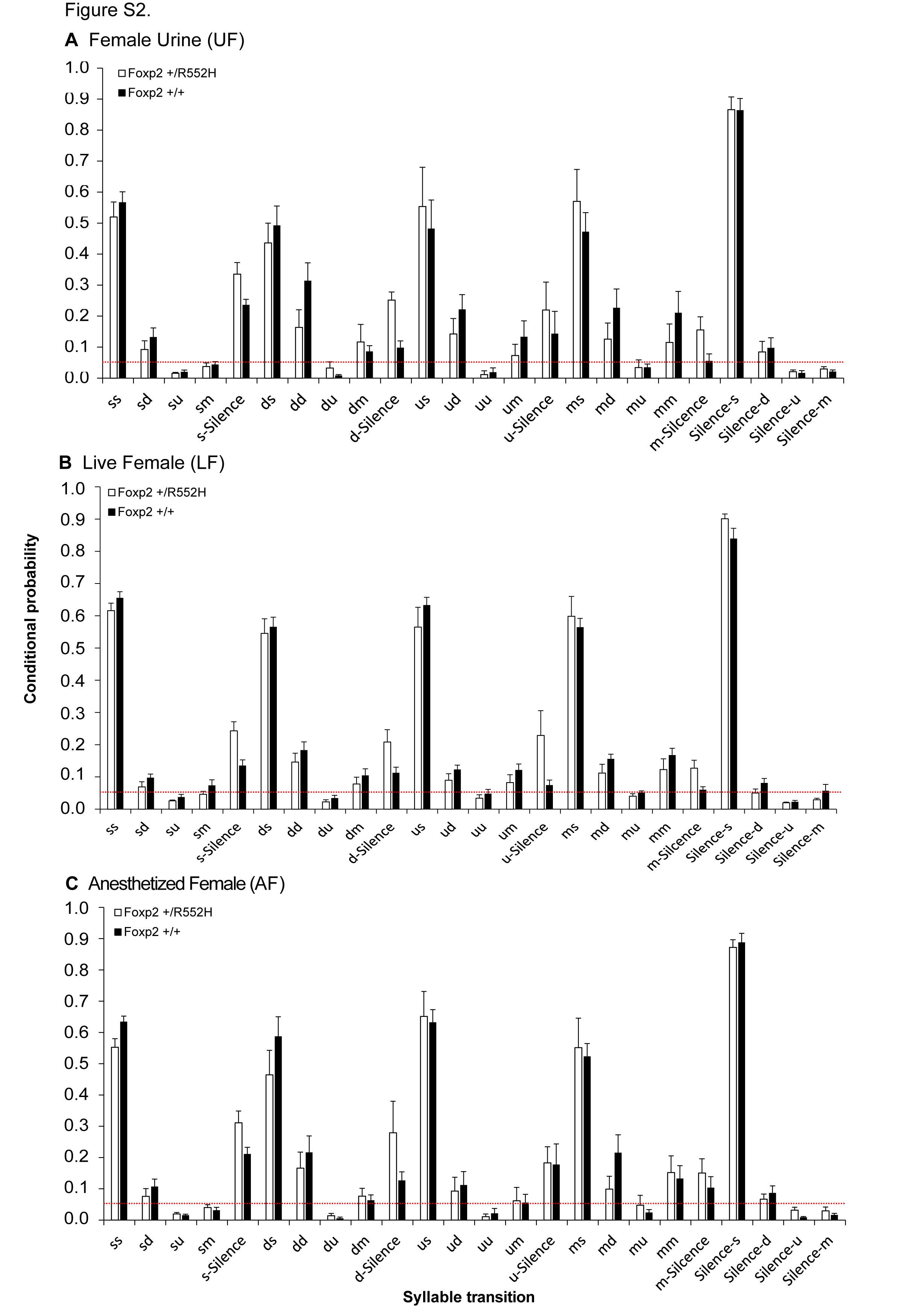

Supplement: Figure S2 — Conditional probabilities for each transition type across context. (A) UF; (B) LF; (C) AF contexts for wildtypes (n = 8) and Foxp2-R552H heterozygous (n = 10) mice. Red dashed lines indicate the 0.05 threshold for values used to generate the syntax graphs. For statistical comparisons between genotype for each transition type see values in Figures 6D–F. [file Image2.JPEG]

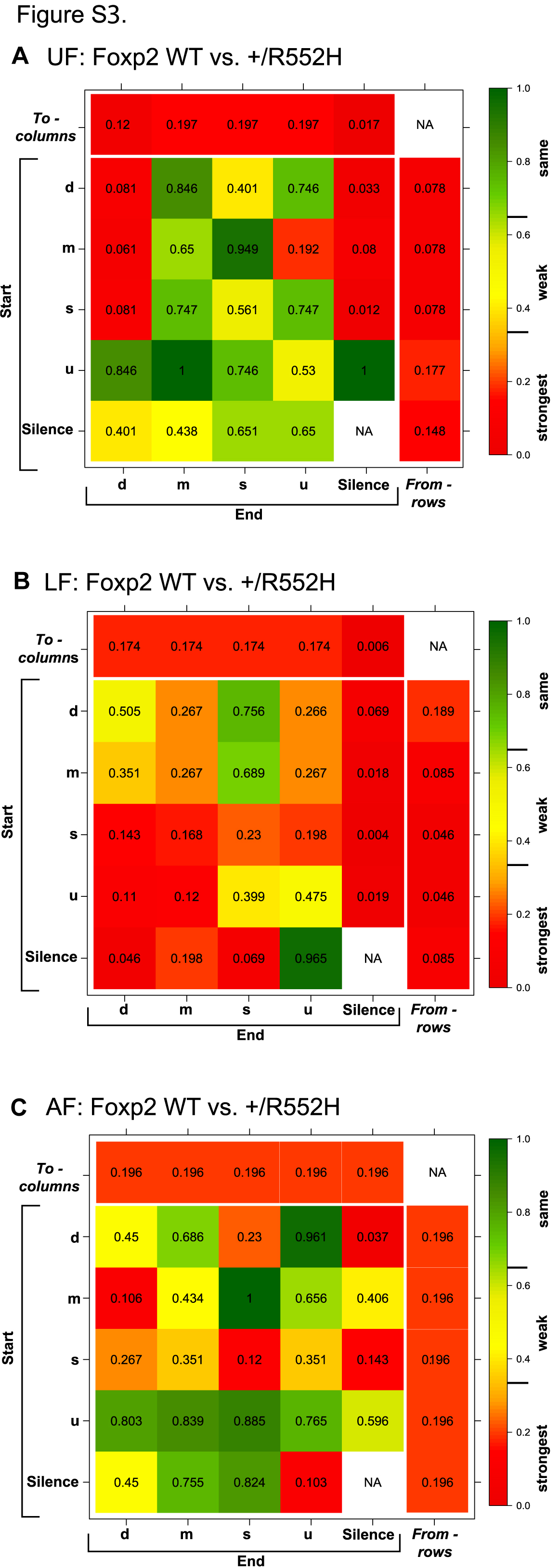

Supplement: Figure S3 — Statistical analyses of syntax with Benjamini-Hochberg correction. Shown are the Benjamini-Hochberg analyses of the data in Figures 6D–F. [file Image3.JPEG]

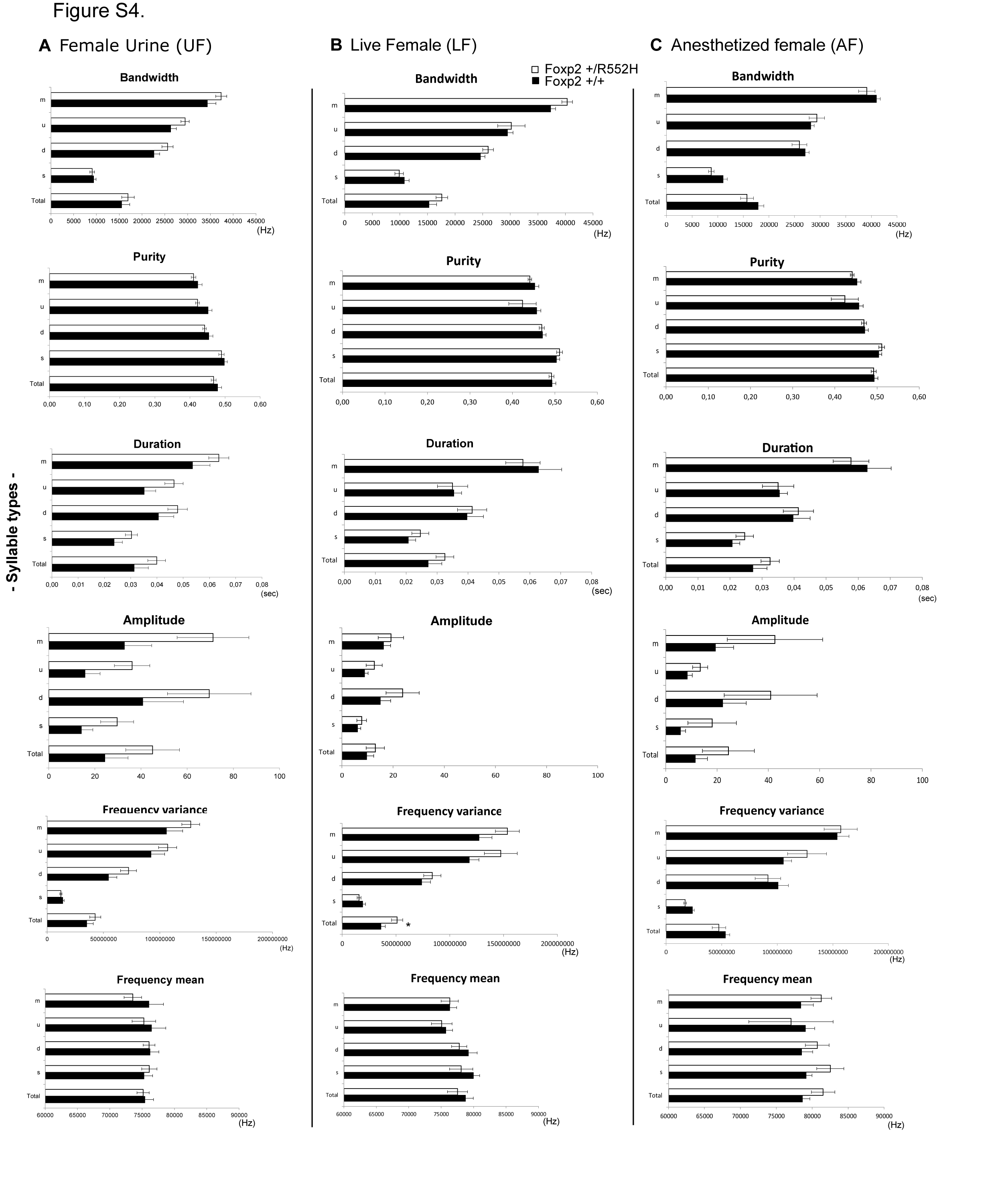

Supplement: Figure S4 — Acoustic features measured in each context replicate experiment. Data are presented as mean ± SEM. *p < 0.05 using Wilcoxon-Mann-Whitney tests for independent samples (n = 15 WT; 16 heterozygous males). Explanation for other items is the same as Figure S1. [file Image4.JPEG]

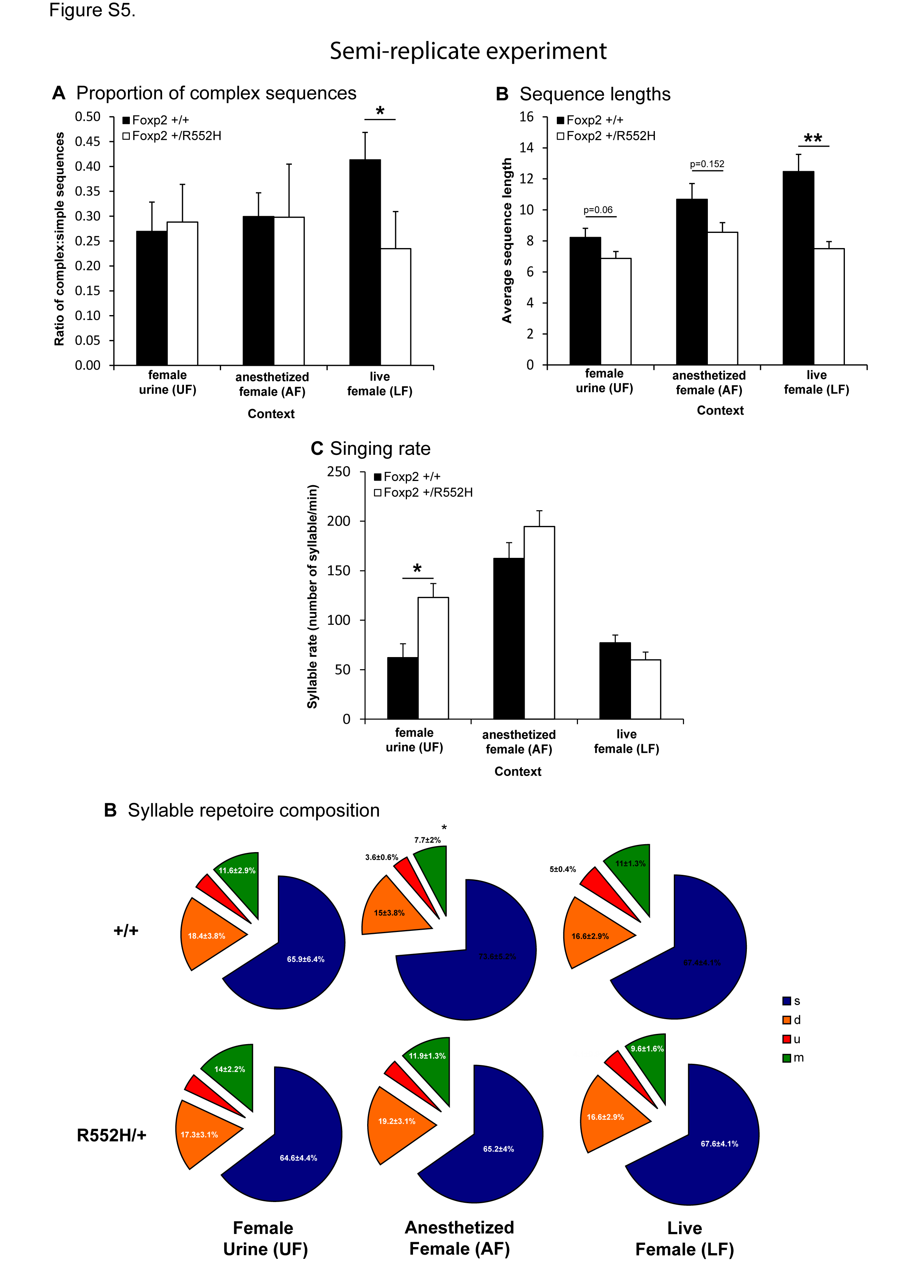

Supplement: Figure S5 — Syllable sequence and syllable repertoire measures replicate experiment. (A) Ratio of complex song syllable sequences over simple songs in each context. (B) Syllable sequences lengths. (C) Syllable production rate. (D) Repertoire compositions of the four major syllable categories. Data are presented as mean ± SEM. *p < 0.05 using Wilcoxon-Mann-Whitney tests for independent samples (n = 15 WT; 16 heterozygous males). Explanations for other items are the same as Figures 2A,B, 5C,D. [file Image5.JPEG]

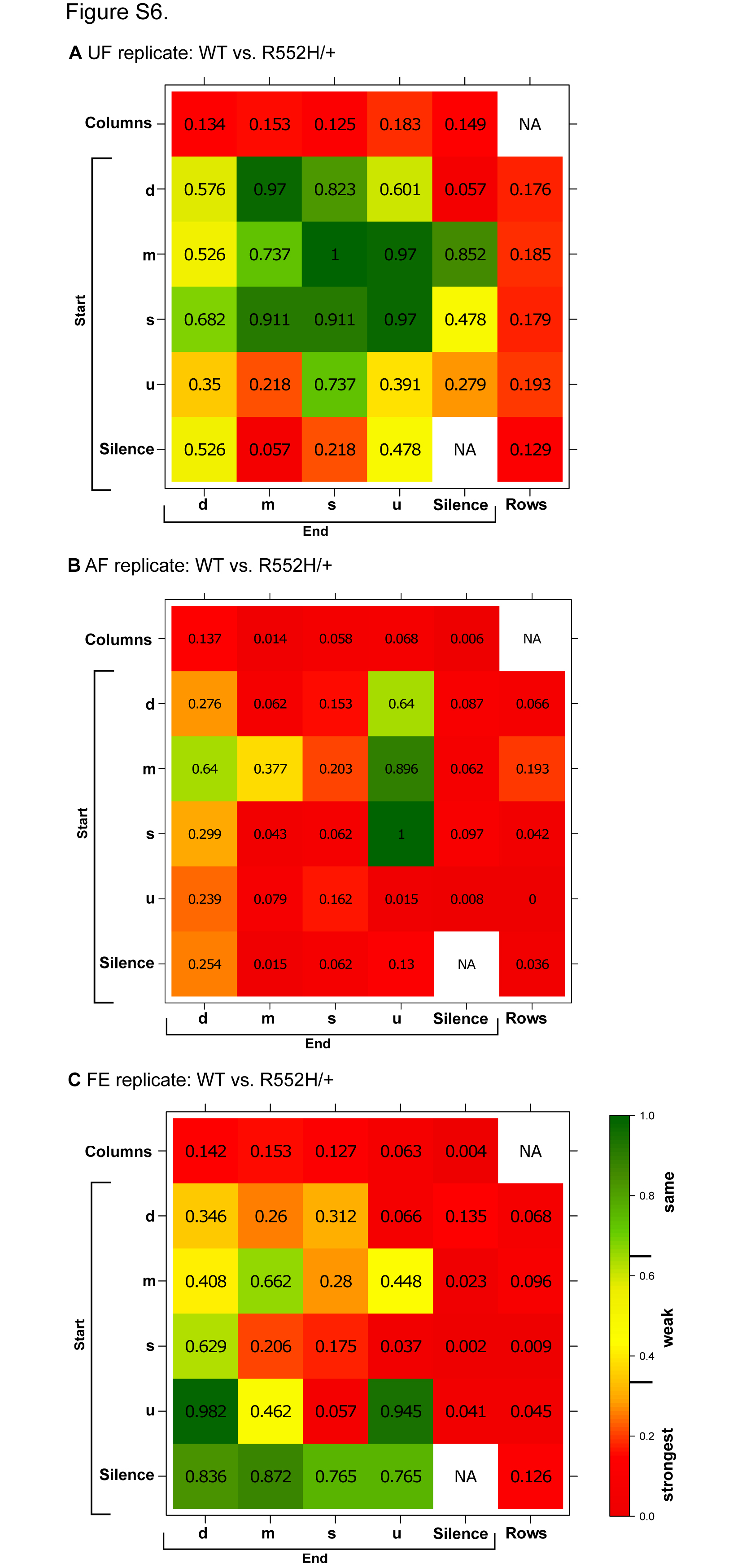

Supplement: Figure S6 — Syntax analyses in the replicate experiment. Explanation the same as Figure 6 (n = 15 WT; 16 heterozygous males). [file Image6.JPEG]

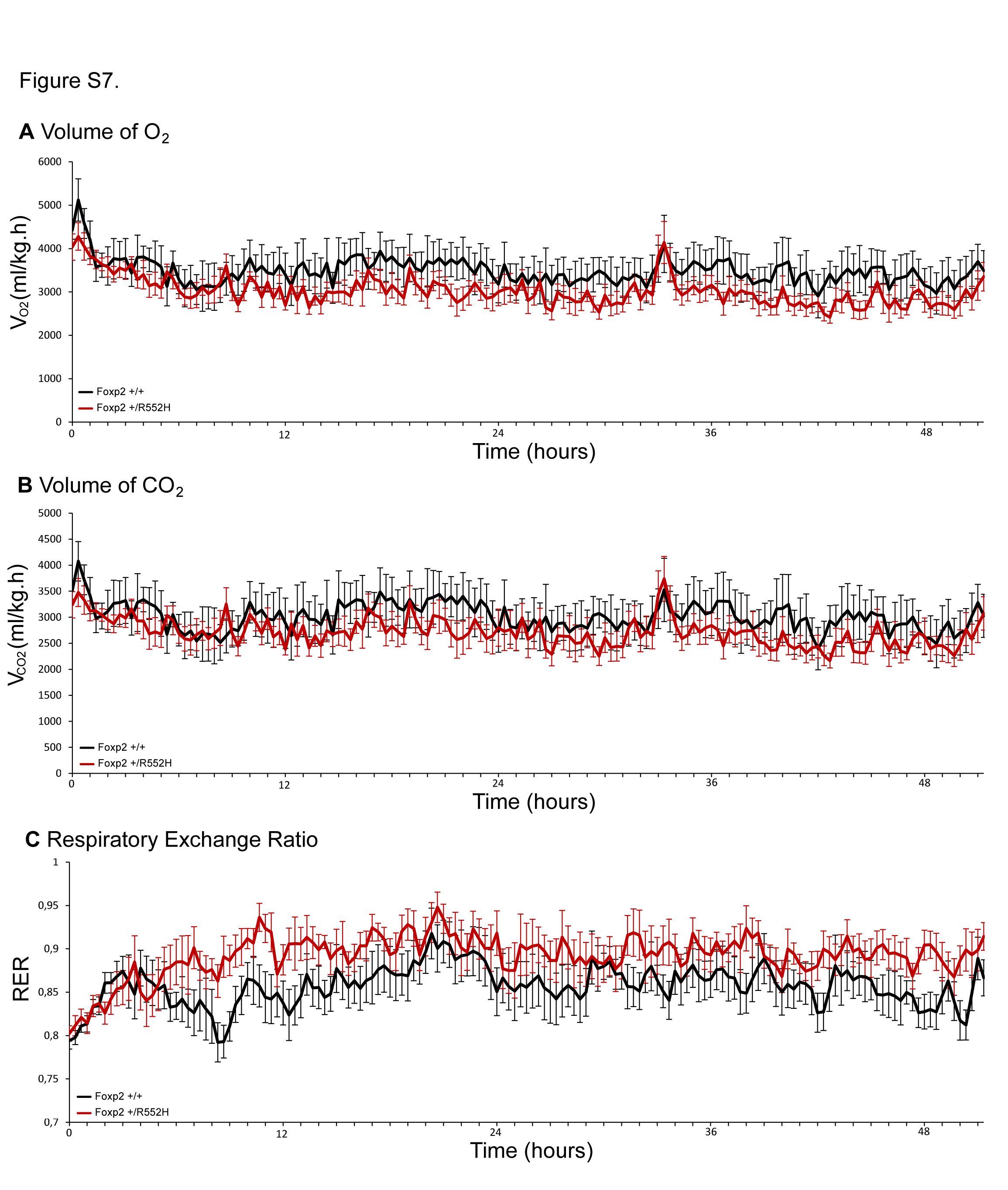

Supplement: Figure S7 — Respiratory analyses. (A) VO2 readings. (B) VCO2 readings. (C) RER = VO2:VCO2 respiratory ratio. There are no statistical differences between respiration (y axes) between wildtype (n = 8) and Foxp2-R552H heterozygous (n = 9) males in the curves. Measurements were recorded every 20 min over a ~48 h period (x-axes) in CLAMS chambers. [file Image7.JPEG]
