# Supplementary material for: A Foxp2 Mutation Implicated in Human Speech Deficits Alters Sequencing of Ultrasonic Vocalizations in Adult Male Mice
Source: Front Behav Neurosci. 2016 Oct 20;10:197. doi: 10.3389/fnbeh.2016.00197 (PMC5071336; doi:10.3389/fnbeh.2016.00197)
Supplement: Table S1 — Raw p-values of the difference between wildtype and Foxp2-R552H/+ mutant mice for each transition type conditional probability (rows to columns) across contexts using Benjamini-Hochberg (FDR) or Bonferroni (FWER) correction. Red, significant at p < 0.05. [file Table1.docx]

**Table S1**: Raw p-values of the difference between wildtype and *Foxp2-R552H*/+ mutant mice for each transition type conditional probability (rows to columns) across contexts using Benjamini-Hochberg (FDR) or Bonferroni (FWER) correction. Red, significant at p < 0.05

| **Condition** | **Variable** | **p-values** | **Benjamini-Hochberg p-values** | **Bonferroni p-values** |
| --- | --- | --- | --- | --- |
| UF | Column "d" | 0.0482 | 0.1205 | 0.241 |
| UF | Column "m" | 0.1661 | 0.1966 | 0.831 |
| UF | Column "s" | 0.1489 | 0.1966 | 0.745 |
| UF | Column "u" | 0.1966 | 0.1966 | 0.983 |
| UF | Column "Silence" | 0.0034 | **0.017** | **0.017** |
| UF | Row "d" | 0.0469 | 0.078 | 0.235 |
| UF | Row "m" | 0.0409 | 0.078 | 0.205 |
| UF | Row "s" | 0.0252 | 0.078 | 0.126 |
| UF | Row "u" | 0.177 | 0.177 | 0.885 |
| UF | Row "Silence" | 0.1182 | 0.148 | 0.591 |
| AF | Column "d" | 0.1425 | 0.1958 | 0.713 |
| AF | Column "m" | 0.1587 | 0.1958 | 0.794 |
| AF | Column "s" | 0.1958 | 0.1958 | 0.979 |
| AF | Column "u" | 0.1877 | 0.1958 | 0.939 |
| AF | Column "Silence" | 0.0883 | 0.1958 | 0.442 |
| AF | Row "d" | 0.1245 | 0.1958 | 0.623 |
| AF | Row "m" | 0.1958 | 0.1958 | 0.979 |
| AF | Row "s" | 0.096 | 0.1958 | 0.480 |
| AF | Row "u" | 0.1756 | 0.1958 | 0.878 |
| AF | Row "Silence" | 0.1339 | 0.1958 | 0.670 |
| LF | Column "d" | 0.0884 | 0.1738 | 0.442 |
| LF | Column "m" | 0.1059 | 0.1738 | 0.530 |
| LF | Column "s" | 0.1738 | 0.1738 | 0.869 |
| LF | Column "u" | 0.1697 | 0.1738 | 0.849 |
| LF | Column "Silence" | 0.0013 | **0.0065** | **0.007** |
| LF | Row "d" | 0.1894 | 0.1894 | 0.947 |
| LF | Row "m" | 0.0683 | 0.085375 | 0.342 |
| LF | Row "s" | 0.0144 | **0.04625** | 0.072 |
| LF | Row "u" | 0.0185 | **0.04625** | 0.093 |
| LF | Row "Silence" | 0.0585 | 0.085375 | 0.293 |
