# Supplementary material for: A Foxp2 Mutation Implicated in Human Speech Deficits Alters Sequencing of Ultrasonic Vocalizations in Adult Male Mice
Source: Front Behav Neurosci. 2016 Oct 20;10:197. doi: 10.3389/fnbeh.2016.00197 (PMC5071336; doi:10.3389/fnbeh.2016.00197)
Supplement: Supplementary file 10 [file DataSheet1.PDF]

Appendix A: Comparison of Syntax Dynamics

For $m = 1, 2$, $i = 1, \ldots, n_m; j \in K; k \in K(j)$; $c \in C$ and $t = 1, \ldots, T_{i,c}^{(m)}$, let $Y_{i,c,t}^{(m)} = k$ if the ith mouse from the mth genotype at the tth time point under the cth context is at state k. Let $\mathbf{Y}_{i,c}^{(m)} = \{Y_{i,c,1}^{(m)}, \ldots, Y_{i,c,T_{i,c}^{(m)}}^{(m)}\}$. We assume a Markov chain framework for the transition dynamics. We follow the usual convention and condition on the initial observations.

The likelihood function is then given by

$$L(\{\mathbf{Y}_{i,c}^{(m)}\}_{m=1,i=1,c\in C}^{2,n_m}, \{\pi_{k|j(i,c)}^{(m)}\}_{m=1,i=1,c\in C,j\in K,k\in K(j)}^{2,n_m}) = \prod_{c\in C}\prod_{m=1}^{2}\prod_{i=1}^{n_m}\prod_{j\in K}\prod_{k\in K(j)} \{\pi_{k|j(i,c)}^{(m)}\}^{n_{k|j(i,c)}^{(m)}},$$

where $\pi_{k|j(i,c)}^{(m)} = \Pr(Y_{i,c,t}^{(m)} = k \mid Y_{i,c,t-1}^{(m)} = j)$ for all $m = 1, 2$, $i = 1, \ldots, n_m$ and all $t = 2, \ldots, T_{i,c}^{(m)}$; $n_{k|j(i,c)}^{(m)} = \sum_{t=2}^{T_{i,c}^{(m)}} 1\{Y_{i,c,t-1}^{(m)} = j, Y_{i,c,t}^{(m)} = k\}$, the total number of transitions from syllable j to syllable k under context c. The maximum likelihood estimates (mle) of $\widehat{\pi}_{k|j(c)}^{(m)}$ are given by $\widehat{\pi}_{k|j(i,c)}^{(m)} = n_{k|j(i,c)}^{(m)} / \sum_{k\in K(j)} n_{k|j(i,c)}^{(m)}$.

Specifically, we have $K = \{d, m, s, u, x\}$, where for notational convenience we represent the state 'silence' by 'x'; $K(j) = K$ for $j = \{d, m, s, u\}$ but $K(x) = \{d, m, s, u\}$; and $C = \{UR, FE, AF\}$. $K(x)$ is different from other $K(j)'s$, since by the nature of the experiment, there is no self transition from x to itself.

	d	m	s	u	x	
d	$\pi_{d\|d}$	$\pi_{m\|d}$	$\pi_{s\|d}$	$\pi_{u\|d}$	$\pi_{x\|d}$	1
m	$\pi_{d\|m}$	$\pi_{m\|m}$	$\pi_{s\|m}$	$\pi_{u\|m}$	$\pi_{x\|m}$	1
s	$\pi_{d\|s}$	$\pi_{m\|s}$	$\pi_{s\|s}$	$\pi_{u\|s}$	$\pi_{x\|s}$	1
u	$\pi_{d\|u}$	$\pi_{m\|u}$	$\pi_{s\|u}$	$\pi_{u\|u}$	$\pi_{x\|u}$	1
x	$\pi_{d\|x}$	$\pi_{m\|x}$	$\pi_{s\|x}$	$\pi_{u\|x}$	−	1

Table 1: The transition probabilities (sans the indicators (i, c) for mouse and context).

1 Comparison between Genotypes

The likelihood function under the context c is then given by

$$L(\{\mathbf{Y}_{i,c}^{(m)}\}_{m=1,i=1,c\in C}^{2,n_m}, \{\pi_{k|j(i,c)}^{(m)}\}_{m=1,i=1,c\in C,j\in K,k\in K(j)}^{2,n_m}) = \prod_{c\in C}\prod_{m=1}^{2}\prod_{i=1}^{n_m}\prod_{j\in K}\prod_{k\in K(j)} \{\pi_{k|j(i,c)}^{(m)}\}^{n_{k|j(i,c)}^{(m)}}.$$

For each mouse i from the mth genotype, let the probability of making a transition from state j to state k under context c be distributed as $F_{k|j(c)}^{(m)}$. That is, $\pi_{k|j(i,c)}^{(m)} \sim F_{k|j(c)}^{(m)}$. In Table 1 above, the cells within each column correspond to transitions *to* that column syllable from different row syllables, and the cells within each row correspond to transitions *from* that row

syllable to different column syllables. Our goal is to test if the context-specific transition probabilities *to* and *from* particular syllables differ significantly between the two genotypes of mice. We formulate these problems as testing the joint null hypotheses

$$H_{0,k|j(c)} : F^{(1)}_{k|j(c)} = F^{(2)}_{k|j(c)} \text{ for all pairs } (j,k) \in \mathcal{S}$$
$$\text{against} \quad H_{1,k|j(c)} : F^{(1)}_{k|j(c)} <_{st} F^{(2)}_{k|j(c)} \text{ or } F^{(2)}_{k|j(c)} <_{st} F^{(1)}_{k|j(c)} \text{ for some pair } (j,k) \in \mathcal{S}.$$

The set $\mathcal{S}$ depends upon the syllable and type of transitions (*to* or *from*, column or row) we are interested in. For example, if interest lies in the transition probabilities *from* the syllable d to different syllables, we look at the corresponding row and $\mathcal{S} = \{(j,k) : j = d; k = d, m, s, u, x\}$. Similarly, if interest lies in the transition probabilities *to* the syllable d from different syllables, we look at the corresponding column and $\mathcal{S} = \{(j,k) : j = d, m, s, u, x; k = d\}$, and so on. Here $F^{(1)} <_{st} F^{(2)}$ denotes that $F^{(2)}$ is stochastically larger than $F^{(1)}$. Under $H_{0,k|j(c)}$, when there is no difference between the distributions $F^{(1)}_{k|j(c)}$ and $F^{(2)}_{k|j(c)}$, the probability of some $\pi^{(1)}_{k|j(i_1,c)}$ from the first genotype exceeding an $\pi^{(2)}_{k|j(i_2,c)}$ from the second genotype will equal the probability of $\pi^{(2)}_{k|j(i_2,c)}$ from the second genotype exceeding $\pi^{(1)}_{k|j(i_1,c)}$ from the first genotype. That is, $\Pr(\pi^{(1)}_{k|j(i_1,c)} > \pi^{(2)}_{k|j(i_2,c)}) = \Pr(\pi^{(2)}_{k|j(i_2,c)} > \pi^{(1)}_{k|j(i_1,c)})$ for any mouse i_1 of the first genotype and any mouse i_2 of the second genotype. On the other hand, under $H_{1,k|j(c)}$, when one of the distributions $F^{(1)}_{k|j(c)}$ and $F^{(2)}_{k|j(c)}$ is stochastically larger than the other, $\Pr(\pi^{(1)}_{k|j(i_1,c)} > \pi^{(2)}_{k|j(i_2,c)}) \neq \Pr(\pi^{(2)}_{k|j(i_2,c)} > \pi^{(1)}_{k|j(i_1,c)})$ for any mouse i_1 of the first genotype and any mouse i_2 of the second genotype.

We used Wilcoxon-Mann-Whitney (WMW) rank sum tests on the $\pi^{(m)}_{k|j(c)}$'s to test the hypotheses. Since the actual probabilities $\pi^{(m)}_{k|j(i,c)}$ are unknown, we follow the usual convention and work with their mle's, ignoring estimation uncertainty. Suppressing the suffixes in favor of cleaner notation, letting $R^{(m)}_i$ denote the rank of the transition probability of the ith mouse in the pooled sample, with n_1 mice of the first genotype and n_2 of the second, the WMW test statistic for testing $H_0 : F^{(1)} = F^{(2)}$ against $H_1 : F^{(1)} <_{st} F^{(2)}$ or $F^{(2)} <_{st} F^{(1)}$ is given by $U = \sum_{i:m=1} R^{(m)}_i - n_1(n_1+1)/2$. Under $H_0 : F^{(1)} = F^{(2)}$, $R^{(m)}_i$'s will be evenly distributed among the set of possible values $\{1, 2, \ldots, N = n_1 + n_2\}$. Under $H_1 : F^{(1)} <_{st} F^{(2)}$ or $F^{(2)} <_{st} F^{(1)}$, $R^{(1)}_i$'s will tend be smaller or larger than $R^{(2)}_i$'s, on average. Small or large values of U thus indicate departure from the null hypothesis.

We combine the p-values returned by the 'local' tests $H_{0,k|j(c)}$ to obtain a test for the joint null $H_{0(c)}(\mathcal{S}) = \cap_{(j,k)\in\mathcal{S}} H_{0,k|j(c)}$. Several approaches of combining local p-values have been proposed in the literature. See, for example, Fisher (1932), Stouffer, et al. (1949) and Edgington (1972). We used the truncated product method (TPM) of Zaykin, et al. (2002) which is more robust to the presence of a few large p-values. Based on L local p-values p_i, the test statistic for the global test is defined as $T(\tau) = \prod_{i=1}^{L} p_i^{1\{p_i<\tau\}}$, where $1\{\cdot\}$ is the indicator function and τ is a truncation limit. Zaykin, et al. (2002) used $\tau = 0.05$, Neuhäuser and Bretz (2005) $\tau = 0.50$ and Neuhäuser (2003) $\tau = 0.70$. Sheng and Yang

(2013) proposed evaluating $T(\tau)$ for a set of candidate τ values and using the minimum p-value observed at these candidate truncation points. We used a fine grid of τ values - $\{0.05, 0.06, \ldots, 0.99, 1.00\}$.

Under the null, the p-values are marginally uniformly distributed. If the dependence among the local tests is ignored, the null distribution of $T(\tau)$ for each candidate value of τ can be estimated using samples of uniformly distributed p_i's. To account for the dependence among the local tests, we used a permutation based Monte Carlo procedure instead. Each local null hypothesis postulates that the two genotypes of mice share a common distribution for the corresponding transition probabilities. Any evidence to the contrary can be destroyed by reallocating the mice randomly to the two genotypes. Each such occasion generates a sample of 24 local p-values for the 24 local WMW tests which can then be combined to obtain a Monte Carlo sample of the TPM based test statistics for the transition probabilities *to* and *from* the five syllables. For each truncation limit τ, the p-values of the combined tests are then estimated as the proportions of samples in which the observed $T(\tau)$'s are less than the resampled $T(\tau)$ values.

Tests for differences in the global dynamics, where $\mathcal{S} = \{(j,k) : j \in K, k \in K(j)\}$, can be similarly constructed.

We used R to implement our method. Many other common statistical softwares allow routine implementation of WMW tests. Estimates of the null distributions of the TPM based test statistics were based on 10,000 random reallocations of the mice. The p-values for local tests as well as the TPM based tests for different rows and columns are presented in Figure 6 in the main paper. See the main paper also for a discussion of these results.

2 Comparison between Contexts

The likelihood function for the genotype m is given by

$$L(\{\mathbf{Y}_{i,c}^{(m)}\}_{i=1,c\in C}^{n_m}, \{\pi_{k|j(i,c)}^{(m)}\}_{i=1,c\in C,j\in K,k\in K(j)}^{n_m}) = \prod_{c\in C}\prod_{i=1}^{n_m}\prod_{j\in K}\prod_{k\in K(j)} \{\pi_{k|j(i,c)}^{(m)}\}^{n_{k|j(i,c)}^{(m)}}.$$

Here our goal is to test if the mice from a given genotype m can alter the transition probabilities *to* and *from* particular syllables significantly between different pairs of contexts c_1, c_2. We again formulate these problems as testing the joint null hypotheses

$$H_{0,k|j}^{(m)} : F_{k|j(c_1)}^{(m)} = F_{k|j(c_2)}^{(m)} \text{ for all pairs } (j,k) \in \mathcal{S}$$
$$\text{against} \quad H_{1,k|j}^{(m)} : F_{k|j(c_1)}^{(m)} <_{st} F_{k|j(c_2)}^{(m)} \text{ or } F_{k|j(c_1)}^{(m)} <_{st} F_{k|j(c_2)}^{(m)} \text{ for some pair } (j,k) \in \mathcal{S}.$$

Since the comparison here involve the same set of mice from a fixed genotype, we used Wilcoxon signed rank (WSR) tests on the $\pi_{k|j(c)}^{(m)}$'s, and not WMW tests. The rest of the

testing procedure is very similar to that described in Section 1 of this Appendix. See also the main paper for a discussion of the results.

References

Edgington, E. S. (1972). An additive method for combining probability values from independent experiments. *Journal of Psychology*, 80, 351-363.

Fisher, R. A. (1932). *Statistical Methods for Research Workers*. Oliver and Boyd, London.

Mann, H. B. and Whitney, D. R. (1947). On a test of whether one of two random variables is stochastically larger than the other. Annals of Mathematical Statistics, 18, 50-60.

Neuäuser, M. Tests for genetic differentiation. (2003). *Biometrical Journal*, 974-984.

Neuhäuser, M. and Bretz, F. (2005). Adaptive designs based on the truncated product method. *BMC Medical Research Methodology*, 5.

Sheng, X. and Yang, J. (2013). An adaptive truncated product method for combining dependent p-values. *Economics Letters*, 112, 180-182.

Stouffer, S. A., Suchman, E. A., DeVinney, L. C., Star, S. A. and Williams Jr., R. M. (1949). *The American Soldier - Adjustment During Army Life*. Princeton Univ. Press, Princeton.

Zaykin, D. V., Zhivotovsky, L. A., Westfall, P. H. and Weir, B. S. (2002). Truncated product method for combining p-values. Genetic Epidemiology, 22, 170-185.
